# Determining direct binders of the Androgen Receptor using a high-throughput Cellular Thermal Shift Assay

**DOI:** 10.1038/s41598-017-18650-x

**Published:** 2018-01-09

**Authors:** Joseph Shaw, Mathew Leveridge, Charlotta Norling, Jakob Karén, Daniel Martinez Molina, Daniel O’Neill, James E. Dowling, Paul Davey, Suzanna Cowan, Michael Dabrowski, Martin Main, Davide Gianni

**Affiliations:** 1 Discovery Sciences, Innovative Medicines and Early Development Biotech Unit, AstraZeneca, 310 Cambridge Science Park, Cambridge, UK; 2 Oncology, Innovative Medicines and Early Development Biotech Unit, AstraZeneca, 35 Gatehouse Park, Waltham, MA USA; 3 Oncology, Innovative Medicines and Early Development Biotech Unit, AstraZeneca, 310 Cambridge Science Park, Cambridge, UK; 4 Pelago Bioscience AB, 171 65 Solna, Sweden

## Abstract

Androgen Receptor (AR) is a key driver in prostate cancer. Direct targeting of AR has valuable therapeutic potential. However, the lack of disease relevant cellular methodologies capable of discriminating between inhibitors that directly bind AR and those that instead act on AR co-regulators has made identification of novel antagonists challenging. The Cellular Thermal Shift Assay (CETSA) is a technology enabling confirmation of direct target engagement with label-free, endogenous protein in living cells. We report the development of the first high-throughput CETSA assay (CETSA HT) to identify direct AR binders in a prostate cancer cell line endogenously expressing AR. Using this approach, we screened a pharmacology library containing both compounds reported to directly engage AR, and compounds expected to target AR co-regulators. Our results show that CETSA HT exclusively identifies direct AR binders, differentiating them from co-regulator inhibitors where other cellular assays measuring functional responses cannot. Using this CETSA HT approach we can derive apparent binding affinities for a range of AR antagonists, which represent an intracellular measure of antagonist-receptor Ki performed for the first time in a label-free, disease-relevant context. These results highlight the potential of CETSA HT to improve the success rates for novel therapeutic interventions directly targeting AR.

## Introduction

A key requirement for a small molecule drug to exert a pharmacological effect is to bind with sufficient affinity and duration to its target protein. Despite this, few options are available to directly measure a compound binding to a protein within the more complex cellular and *in vivo* systems to which they will ultimately be applied^1^. As such, the suitability of a molecule for progression as a drug is frequently assessed from indirect functional cellular responses, which can be influenced by interactions with co-regulators and components of associated signalling pathways. In recent years, the absence of direct target engagement technologies has manifested as clinical failure of drugs which have not demonstrated conclusive evidence of binding to the intended target^2^.

The low success rate of translating an early drug discovery program into clinical efficacy has led to increased focus on the disease-relevance of screening assays. For cell-based assays, recent reports highlight the drive towards primary cell types, endogenous target expression and label-free systems^3–6^. Hence there is an urgent need for cellular assays which measure direct target engagement in a disease-relevant setting, enabling more predictive translation into clinical efficacy. The Cellular Thermal Shift Assay (CETSA^®^) is a technology capable of fulfilling these requirements. It relies on the inherent thermal stability of the target protein within the cell, and a change in thermal stability induced upon compound binding^7^.

Androgen Receptor (AR) is a well-validated target for the treatment of prostate cancer and a key driver of castration resistant prostate cancer (CRPC)^8^. AR is a nuclear hormone receptor which responds to androgens by undergoing a conformational change and translocating to the nucleus where it acts as a transcription factor to modulate gene expression^9,10^. AR is modular in structure and is composed of a N-terminal domain, a DNA-binding domain and a ligand-binding domain^9,11^, against which several small molecule inhibitors have been developed^8,12^. AR’s role in driving prostate cancer was defined following the observation that androgen starvation by castration can halt disease progression. In the majority of cases however, relapse to CRPC is observed, a process reliant on AR-driven transcription^13^. A range of AR antagonists are in development or approved to treat CRPC, but are hampered by resistance through amplification, truncation or single nucleotide polymorphisms within the AR gene^12^. Novel AR antagonists able to overcome resistance may offer new, much-needed therapies to treat CRPC.

Current cellular assay technologies typically measure changes in the functional consequence of AR agonism, namely transcription of androgen-responsive genes. However, AR-driven transcription is influenced by co-regulators from a complex network of pathways and interactions. Like other steroid receptors, in the absence of ligand AR is complexed with chaperones including Heat shock protein 90 (Hsp90) and co-chaperones such as p23^10,14,15^. Upon activation AR recruits a variety of proteins including numerous epigenetic regulators which act as part of an AR signalling complex to facilitate modulation of gene transcription. Bromodomain-containing proteins such as BRD4^16^ and ATAD2^17^, and epigenetic regulators such as Enhancer of zeste homologue 2 (EZH2)^18^ and lysine specific demethylase 1 (LSD1)^19^ influence transcription of androgen-responsive genes, while components of the mixed-lineage leukemia (MLL) complex facilitate AR transcriptional activation via an interaction with menin^20^. AR-driven transcription is further modulated by post-translational modification mediated through receptor tyrosine kinase signalling^10^. The variety of factors affecting AR-driven transcription can make interpretation of functional responses difficult^21^, a common problem for many intracellular drug targets.

To facilitate identification of direct AR binders, we sought to apply CETSA to measure direct cellular target engagement. We report the first high-throughput CETSA (CETSA HT) assay for the determination of intracellular target engagement with AR, measured in disease-relevant cell lines derived from primary human prostate cancers. Using this screening-compatible CETSA HT assay we profiled a training set of compounds in parallel with cellular assays measuring AR-driven transcription. We demonstrate the ability of CETSA HT to correlate functional responses with AR target engagement, and more importantly to differentiate cellular responses driven through co-regulators. In addition, we observed distinct AR thermal profiles depending on the receptor ligand occupancy. This enabled ligand competition experiments to determine parameters for apparent intracellular binding affinity. We therefore demonstrate for the first time that CETSA HT can determine intracellular receptor-ligand binding affinity, using label-free compounds, with receptor expressed in its endogenous context within a prostate cancer cell line.

The ability to confidently identify direct AR binders, and to derive physiologically relevant measures of intracellular binding affinity, has the potential to expedite the identification of novel and improved AR antagonists for the treatment of CRPC. This method might have broad application in drug discovery.

## Results

### Distinct cellular thermal stability of agonist- and antagonist-bound Androgen Receptor

CETSA is based on measurements of remaining soluble target protein against a background of thermally denatured and precipitated proteins following a heat challenge^7^. To develop a CETSA assay for AR, we first investigated AR thermostability using quantitative western blot as a readout to detect the amount of soluble thermostable protein following heat shock, a methodology henceforth referred to as CETSA Classics. For these experiments we utilised a monoclonal cell line, CWR22Pc-R1-AD1^22–24^, derived from a primary human prostate cancer, CWR22^25^. This prostate-cancer derived cell line expresses a single copy of AR, resolved as a single species by western blot (Supplementary Fig. S1), and reproduces several key aspects of AR-driven prostate cancer disease, such as phenotypic responses to stimulation with the AR agonist α-Dihydrotestosterone (DHT) as well as treatment with AR antagonists such as Enzalutamide^23^. Previously described CETSA protocols were employed to measure in-cell target engagement as a change in cellular AR thermal stability^7,26^. Briefly, compound treated cells are subjected to a heat shock spanning a range of temperatures using a PCR machine. As the temperature applied to each sample increases, the target protein unfolds and aggregates. The temperature at which this aggregation occurs is a consequence of the intrinsic thermal stability of AR within the cell. Following heat shock, cells are lysed and the soluble thermostable AR fraction and aggregated insoluble AR fraction are physically seperated by centrifugation, allowing quantification of remaining soluble thermostable AR by western blot (CETSA Classics). This allows analysis of changes in the thermal stability of cellular AR following compound addition as evidence of target engagement.

**Figure 1:**
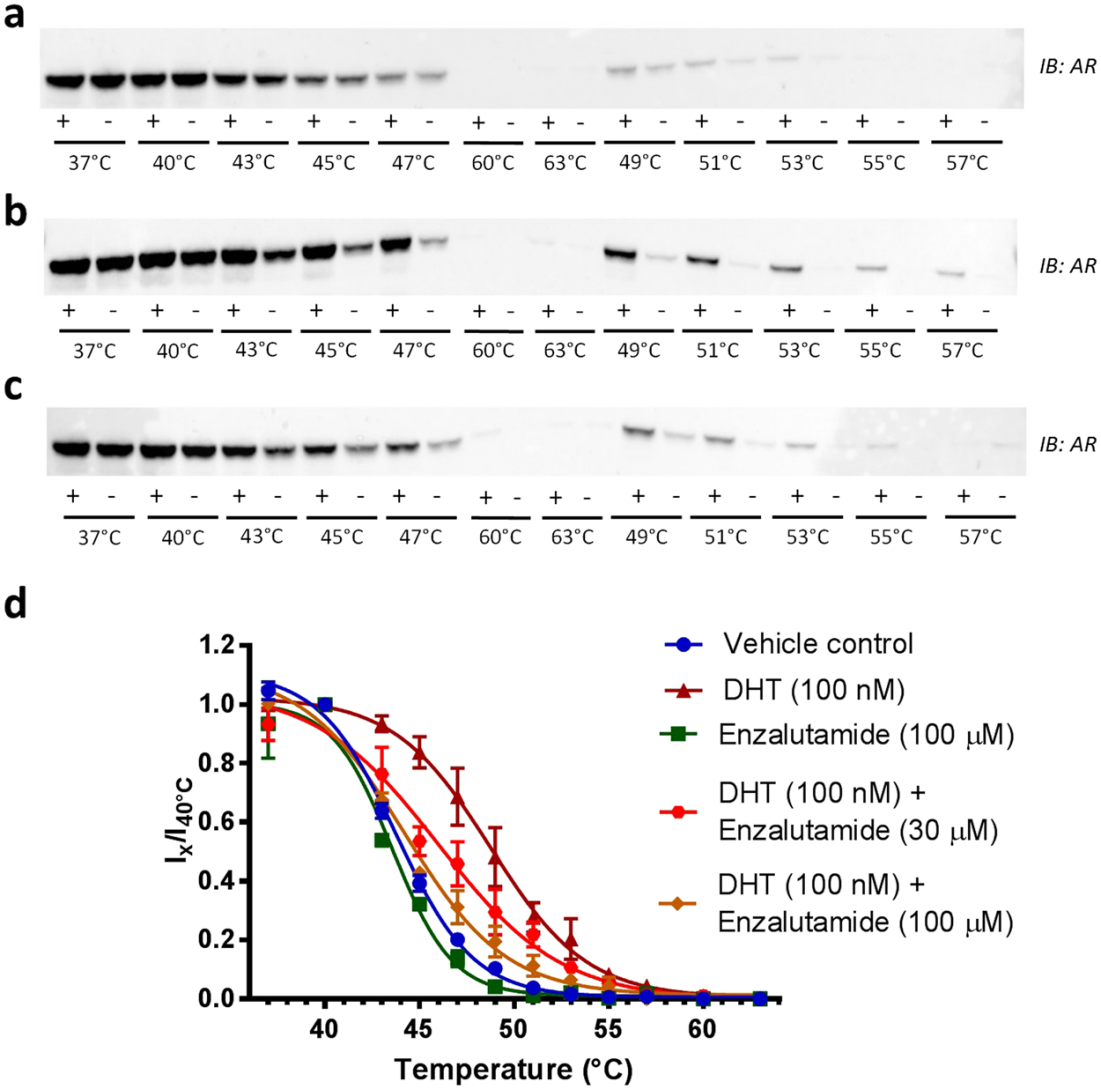
CETSA Classics for in-cell AR target engagement. Representative western blots showing thermostable AR following indicated heat shocks in the presence (+) or absence (−) of (**a**) Enzalutamide (100 µM), (**b**) DHT (100 nM), or (**c**) DHT (100 nM) with Enzalutamide (30 µM). In each case a single cropped western blot is shown. Full-length blots are presented in Supplementary Fig. S11. (**d**) Quantification of thermostable AR western blots from CETSA Classics. Blot intensity (I) is normalised to intensity from the 40 °C sample. Thermal stabilisation was observed upon addition of the agonist dihydrotestosterone (DHT, 100 nM), but not by addition of the antagonist Enzalutamide (100 µM). Co-dosing Enzalutamide with DHT reversed DHT-induced thermal stabilisation in a concentration dependent manner. Data the mean ± SD of n ≥ 3, from ≥3 replicates.

As shown in Fig. 1a, we initially failed to observe any significant change in AR thermal stability upon addition of the antagonist Enzalutamide, despite well documented cellular and clinical activity of Enzalutamide against AR^27^ and mass spectrometry analysis confirming intracellular compound accumulation (Supplementary Fig. S1). However, upon addition of the AR agonist DHT, we observed a clear thermal stabilisation of AR indicative of target engagement (Fig. 1b,d). Given the discrepancy of thermal profiles with two compounds which have both been shown to bind AR at the ligand-binding domain^27,28^, we sought to explore co-dosing of the agonist DHT with the antagonist Enzalutamide. The addition of Enzalutamide was able to reverse the thermal stabilisation caused by DHT (Fig. 1c,d), to our knowledge the first observation of its kind for a CETSA assay. This was evident in a concentration dependent manner, with 30 µM Enzalutamide causing partial reversal of agonist-induced thermal stabilisation, and 100 µM Enzalutamide able to reverse the AR thermal profile from ‘agonist-stabilised’, to a profile similar to that observed for AR in the presence of 100 µM Enzalutamide alone, or for AR in the absence of any ligand (Fig. 1d). The observation of antagonist reversing agonist-mediated AR thermal stabilisation demonstrates for the first time the ability to determine agonist or antagonist binding mode from CETSA experiments.

### Development of a CETSA HT assay to quantify in-cell Androgen Receptor target engagement

Our finding that binding mode might be determined using this competitive CETSA approach pointed to the potential to develop two variations of a CETSA assay to differentially screen for agonist or antagonist binders of AR. As these experiments required a higher degree of throughput than afforded by western blot, we applied a similar methodology enabling high-throughput CETSA (CETSA HT) using AlphaScreen^®^ FRET technology^29^. Panels of mouse and rabbit derived anti-AR antibodies were screened to identify antibody pairs yielding a specific AlphaScreen^®^ signal in CWR22-Pc-R1-AD1 cell lysates upon the addition of anti-mouse AlphaScreen^®^ donor and anti-rabbit AlphaScreen^®^ acceptor beads. Following optimisation of antibody concentrations and cell density (Supplementary Fig. S2), the use of AlphaScreen^®^ to quantify soluble thermostable AR allowed the establishment of a robust (Robust Z’-factor = 0.54–0.74), plate-based CETSA assay compatible with high-throughput applications (Supplementary Fig. S2e).

**Figure 2:**
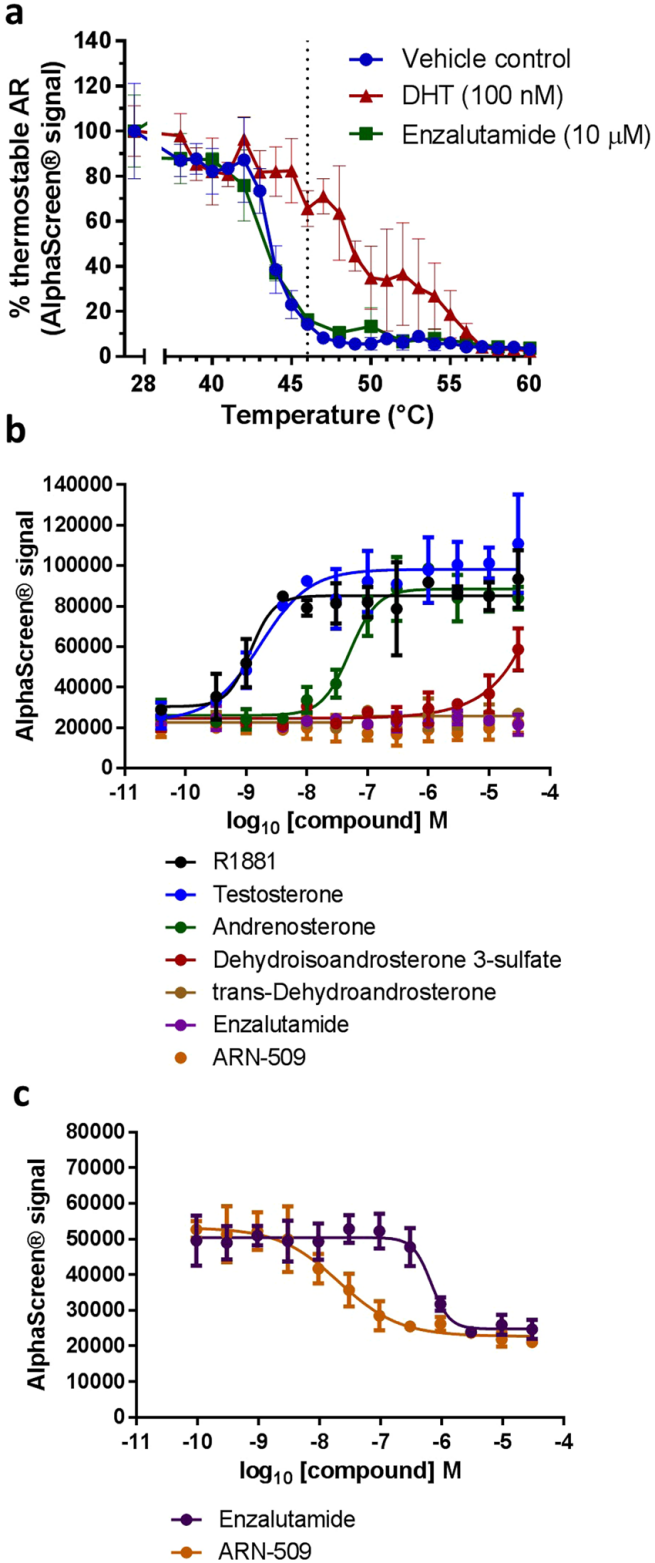
Cellular target engagement of AR agonists or antagonists by CETSA HT. (**a**) An in-cell CETSA HT thermal melt curve confirming thermal stabilisation of AR upon addition of the agonist dihydrotestosterone (DHT), but no stabilisation in the presence of the antagonist Enzalutamide. Data is the mean ± SD of one replicate, n = 4. (**b**) ITDRF_CETSA_ experiments at 46 °C to determine the potency of agonist target engagement. R1881 EC_50_ = 1.2 nM, Testosterone EC_50_ = 1.7 nM, Andrenosterone EC_50_ = 50.6 nM. Data is the mean ± SD of two technical replicates. (**c**) ITDRF_CETSA_ experiments at 46 °C in the presence of 1 nM DHT to determine the potency of antagonist target engagement. Enzalutamide IC_50_ = 666.8 nM, ARN-509 IC_50_ = 20.6 nM. Data is the mean ± SD of one replicate, n = 6.

Using this newly established CETSA HT assay, we looked at the effect of DHT or Enzalutamide on AR thermal stability in CWR22-Pc-R1-AD1 cells. Similar to the observations made by CETSA Classics shown in Fig. 1, we observed AR thermal stabilisation upon the addition of DHT agonist, while the addition of Enzalutamide antagonist did not induce any thermal stabilisation (Fig. 2a). To confirm these observations, the assay format was switched to isothermal dose response fingerprints (ITDRF_CETSA_)^7^, whereby ligand concentration was varied in the presence of a heat shock at 46 °C. This temperature provided a suitable window between unbound and agonist-bound AR where the majority of cellular AR was thermally destabilised in the absence of agonist target engagement (Fig. 2a). In this format, we determined the relative potency of target engagement for a range of AR agonists (Fig. 2b), while a range of AR antagonists all failed to induce any thermal stabilisation at 46 °C (Fig. 2b, Supplementary Fig. S3). This data demonstrates that CETSA HT can be applied to determine the relative potency of target engagement, with the AR agonist testosterone, and a synthetic stable analogue R1881, demonstrating 50% effective concentrations (EC_50_) for in-cell AR target engagement of 1.7 and 1.2 nM respectively. The partial AR agonist Andrenosterone showed reduced potency of target engagement, with an EC_50_ of 50.6 nM. The inactive metabolic products of testosterone, dehydroisoandrosterone 3-sulphate and trans-dehydroandrosterone, showed only partial or no target engagement respectively (Fig. 2b). These observations were consistent in an alternative AR-positive prostate cancer cell line, LNCaP^30^, demonstrating the thermal behaviour of AR described herein is not only specific to CWR22Pc-R1-AD1 cells (Supplementary Fig. S3). Furthermore, a time-course of compound treatment revealed that thermal stabilisation of AR was apparent following 10 minutes incubation with testosterone or R1881, but complete thermal stabilisation required 30 minutes treatment with andrenosterone, suggesting a requirement for intracellular conversion into the active AR agonist. In contrast, no thermal stabilisation was observed at any point following up to 6 hours treatment with a range of antagonists, confirming AR antagonists do not thermally stabilise AR upon target engagement (Supplementary Fig. S4).

Building on our observation that Enzalutamide reverses DHT-mediated AR thermal stabilisation, we used our newly established CETSA HT assay to perform concentration responses of AR antagonists in the presence of a fixed dose of DHT (1 nM) prior to heatshock at 46 °C. In this format, we observed a concentration-dependent destabilisation of agonist-stabilised AR, which then allowed us to rank relative antagonist potency, with the Enzalutamide 50% inhibitory concentration (IC_50_) determined as 666.8 nM and IC_50_ for ARN-509 determined as 20.6 nM (Fig. 2c). These results suggest that while the binding of AR antagonist alone does not cause a significant change in AR thermal stability, competition of the agonist from the AR ligand-binding domain upon addition of antagonist removes the thermostabilising effect of agonist binding. Hence this effect constitutes a proximal measure of antagonist target engagement.

### CETSA HT differentiates direct AR binders from binders of AR co-regulators

Having confirmed that CETSA HT can be applied to determine intracellular target engagement for both agonists and antagonists of AR, we sought to explore whether the use of this newly generated CETSA HT assay can differentiate between direct AR binders and compounds binding AR co-regulators. To this aim, we defined a pharmacology training set of 25 compounds containing 3 small molecules reported to directly engage AR as antagonists, and a range of compound classes targeting AR co-regulator proteins. Within this training set 22 compounds are reported to target various confirmed or tentative AR co-regulators, and as such were considered likely to impact AR-driven transcription, but were not expected to directly engage with AR. The training set was screened using the AR CETSA HT assay in the presence of 1 nM DHT in CWR22Pc-R1-AD1 cells and the results were compared to a functional downstream measure of AR-driven transcription through a luciferase reporter under the control of an Androgen-response promoter element (ARE) (Table 1, Supplementary Table S1). To capture direct AR-dependent transcriptional effects, we carried out compound treatments for 24 hours in ARE-luciferase CWR22Pc-R1-AD1 cells.

**Table 1:** Comparative study for compound activity in an ARE-Luciferase reporter assay for AR-driven transcription and compound activity in CETSA HT. Raw data is reported in Supplementary Table S1.

Compound	Target and expected mode-of-action	AR-driven transcription (ARE-Luciferase), 24 h, IC_50_ (µM)	AR target engagement (CETSA HT), 2 h, IC_50_ (µM)
Enzalutamide	AR antagonist	0.100	0.288
Hydroxyflutamide	AR antagonist	0.135	0.046
MK-2866	AR antagonist	0.170	0.089
MI-136	Inhibitor of AR co-regulator Menin	7.4	>30
MI-503	Inhibitor of AR co-regulator Menin	11.0	>30
Danusertib	Kinase inhibitor with activity against the AR co-regulator TrkA	2.6	>30
Entrectinib	Kinase inhibitor with activity against the AR co-regulator TrkA	11.2	>30
Bayer inhibitor	Kinase inhibitor with activity against the AR co-regulator TrkA	2.8	>30
JQ1	Inhibitor of AR co-regulator BRD4	0.302	>30
OTX-015	Inhibitor of AR co-regulator BRD4	0.214	>30
Onalespib	Inhibitor of AR chaperone Hsp90	0.537	Reduces total AR
Tanespimycin	Inhibitor of AR chaperone Hsp90	3.2	Reduces total AR
NVP-AUY-922	Inhibitor of AR chaperone Hsp90	0.089	Reduces total AR
Niclosamide	AR downregulator	0.955	Reduces total AR
ACS-J9	AR downregulator	>10	>30
Ailanthone	Inhibitor of p23, mediator of AR-Hsp90 interaction	0.087	>30
WNT-974	Inhibitor of Tankyrase component of the WNT signalling pathway	>30	>30
XAV-939	Inhibitor of Porcupine component of the WNT signalling pathway	>30	>30
GSK-126, EPZ-6438, EED-226, A-395, GSK8814	Inhibitors of AR co-regulators EZH2/ATAD2	>30	>30
GSK2879522	Inhibitor of AR co-regulator LSD1	>30	>30
Abiraterone	Inhibitor of CYP17A, a key component of nongonadal androgen synthesis	>30	>30

As expected, our screen identified known AR antagonists Enzalutamide, Hydroxyflutamide and MK-2866 as active in both CETSA HT and functional AR transcriptional assays (Table 1, Supplementary Table S1). Much evidence indicates that these small molecules exert their AR antagonism by direct binding to the AR ligand binding domain^8,27,31–33^ supporting the concept that our CETSA HT agonist competition assay is able to identify direct AR binders. Our comparative analysis was also able to discriminate compounds that are not reported to directly bind AR but instead are inhibitors of AR co-regulators. While several of these compound classes showed activity in functional AR transcriptional assays, all were inactive in CETSA HT, reinforcing the idea that CETSA HT can exclusively detect direct AR binders.

The menin subunit of the mixed-lineage leukemia (MLL) complex has been reported to act as a co-regulator of AR^20^. Consistent with this, the menin inhibitors MI-136 and MI-503 reduced ARE luciferase response in the low-to-mid µM range after a 24 hour treatment (Table 1, Supplementary Table S1). This effect was also confirmed against an endogenous AR-responsive transcript, FKBP5, by RT-qPCR (Supplementary Fig. S5). As expected, CETSA HT screening showed no evidence of direct target engagement with AR for either of these compounds (Table 1).

The receptor tyrosine kinase tropomyosin receptor kinase A (TrkA) was recently identified as an AR-binding partner and is implicated as a co-regulator^34^. Danusertib and Entrectinib, two kinase inhibitors with reported TrkA activity, and a TrkA inhibitor reported by Bayer^35,36^, were active in the ARE-luciferase assay, decreasing DHT-stimulated AR transcriptional responses, but were inactive in CETSA HT (Table 1, Supplementary Table S1, Supplementary Fig. S5). The transcriptional activator BRD4 has been shown to interact with AR, and BRD4 inhibitors show anti-proliferative effects in models of CRPC^16^. We observed partial activity against AR transcriptional responses with the BRD4 inhibitors JQ1 and OTX-015 following 24 hour treatment. These BRD4 inhibitors were inactive in CETSA HT, confirming lack of direct target engagement with AR (Table 1).

Compounds identified as active in both AR-driven transcription assays and CETSA HT included a range of Hsp90 inhibitors and Niclosamide (Supplementary Table S1, Supplementary Fig. S5). The Hsp90 inhibitor Onalespib has been implicated in proteasome-mediated degradation of AR rather than AR antagonism^37^, as has Niclosamide^38^. The use of a no heat-shock control in the absence of exogenous DHT confirmed reduction in total cellular AR rather than reduction of AR thermal stability caused by competition of DHT (Supplementary Fig. S6). These results suggest that these compounds cause rapid AR degradation precluding measures of target engagement. Of note, the kinetics of AR degradation is revealed by time-course experiments (Supplementary Fig. S7). Another reported AR degrader, ACS-J9^39^, showed weak activity in the ARE-luciferase assay, but no significant target engagement or reduction of AR at 2 hours (Table 1, Supplementary Fig. S6), or following up to 6 hours treatment (Supplementary Fig. S7). The p23 inhibitor Ailanthone blocks the AR-Hsp90 interaction leading to AR degradation^14^, and showed potent activity in indirect AR-driven transcription assays, but no direct AR target engagement (Table 1, Supplementary Table S1).

Compounds within the pharmacology set showing no engagement with AR and no effect on AR-driven transcription included inhibitors of components of the WNT signalling pathway implicated in CRPC^40^, a range of inhibitors of the epigenetic regulator EZH2 recently identified as an AR co-regulator^18^, an inhibitor of ATAD2 thought to co-regulate AR alongside EZH2^17^, an inhibitor of the AR co-regulator LSD1^19^ and a CYP17A inhibitor downregulating synthesis of nongonadal AR agonists^41^ (Table 1). Of note, multiple reports indicate that inhibitors of epigenetic modifiers can require treatments for several days before observation of significant cellular effects^42–44^. Internal data confirmed these compounds were active against their respective targets (data not shown). Given that our functional reporter assay was limited to 24 hours to avoid indirect effects on AR-dependent transcription, the lack of AR-dependent transcriptional effects for these inhibitors is not surprising.

In conclusion, the application of a CETSA HT assay, coupled with a control to measure changes in total AR, allowed differentiation of AR transcriptional responses from AR target engagement. Prominent examples from the pharmacology test set included menin, TrkA, p23 and BRD4 inhibitors (Table 1). These compounds have an impact on AR transcriptional responses, potentially via targeting of co-regulators of AR, but do not directly bind to AR as evidenced by inactivity in the CETSA HT assays. As further evidence that these compounds do not directly bind AR, they showed no target engagement measured by CETSA HT at any point following a time-course experiment in which cells were incubated with compound for a range of timepoints from 10 minutes to 6 hours (Supplementary Fig. S7). In contrast, target engagement could be observed with known AR antagonists in the presence of DHT at different time points, allowing kinetic analysis of target engagement. Taken together these observations demonstrate that in a disease-relevant cell line our CETSA HT assay can identify direct AR binders, differentiating them from co-regulator inhibitors where other cellular assays measuring functional responses cannot.

### Application of CETSA HT to determine apparent intracellular Ki

**Figure 3:**
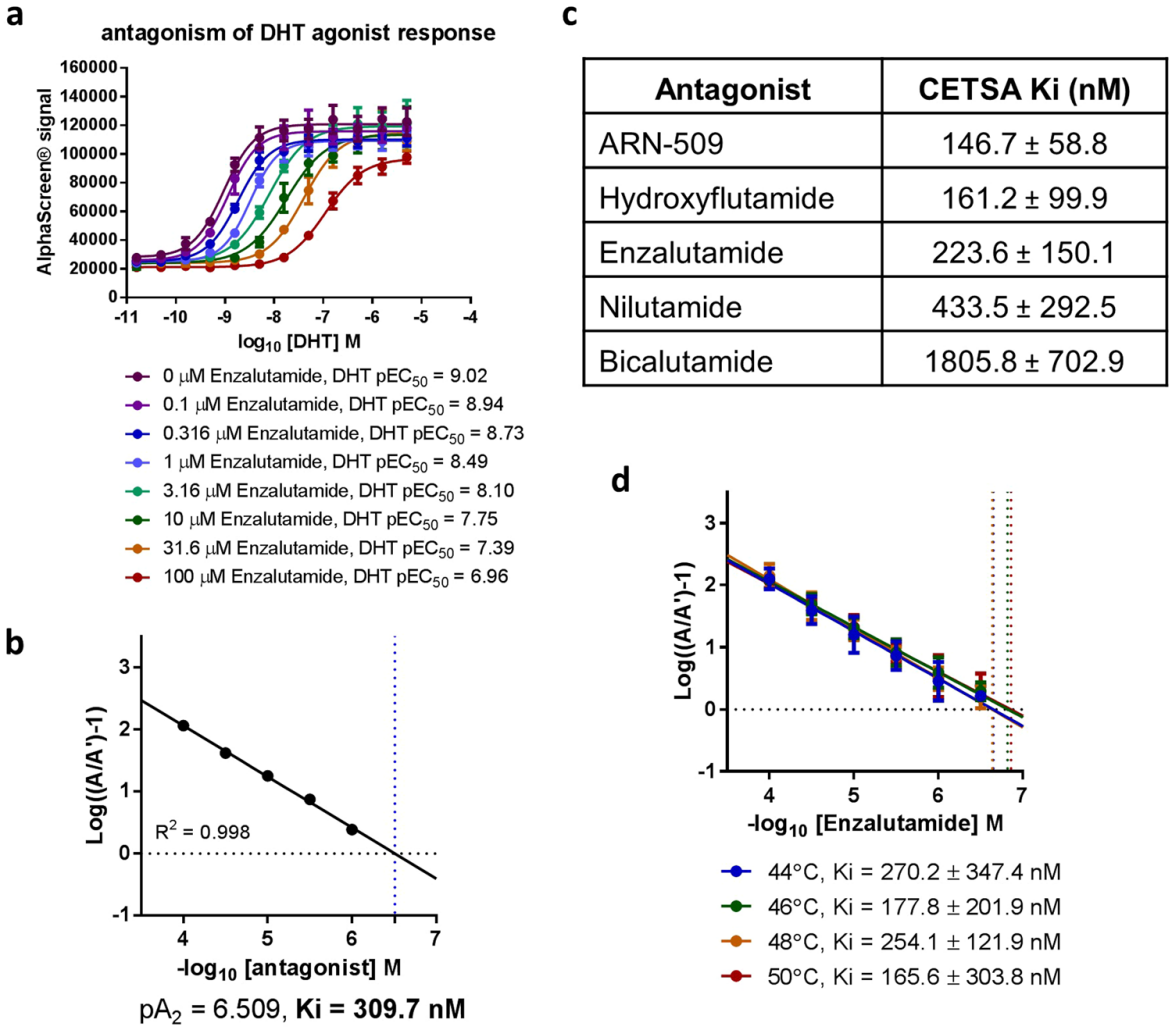
CETSA HT detects antagonism of agonist target engagement to derive apparent intracellular Ki. (**a**) ITDRF_CETSA_ experiments performed for DHT in the presence of increasing concentrations of Enzalutamide. Enzalutamide reverses DHT-mediated thermal stabilisation of AR in a concentration dependent manner, showing competitive antagonism of agonist target engagement. Data shown is the mean ± SD of n = 4 and is representative of five technical repeats, summarised in (**c**). (**b**) Data from (**a**) re-plotted as a Schild plot showing fold change in agonist ITDRF_CETSA_ EC_50_ at indicated antagonist concentration. Data is representative of five technical repeats. (**c**) Intracellular AR antagonist Ki determined by CETSA HT for a range of AR antagonists. Values shown are the geometric mean ± SD of ≥3 technical repeats, each n = 4. Representative raw data is reported in Supplementary Fig. S8. (**d**) Application of CETSA HT to determine intracellular Ki is not significantly affected by the temperature of the heat shock. The plotted data is the mean ± SD of three technical repeats, each n = 4. Representative raw data is reported in Supplementary Fig. S9.

In systems where competitive antagonism is observed, the competition of agonist and antagonist for a common binding pocket is routinely used to derive binding affinity parameters such as the inhibitory constant, Ki. Given the competitive nature of antagonists in our AR CETSA HT assay, we set out to perform more comprehensive competition experiments in this system. While CETSA relies on an irreversible thermal aggregation of the target protein, and is therefore not a system at equilibrium, we attempted to derive an apparent intracellular binding affinity, measured by quantification of target engagement within live cells. To this end, CWR22Pc-R1-AD1 cells were treated with increasing concentrations of the agonist DHT in the presence of various concentrations of Enzalutamide prior to a 46 °C heat shock and quantification of thermostable AR by AlphaScreen^®^. As expected, DHT caused a concentration dependent increase in thermostable AR, demonstrating target engagement with an EC_50_ of 0.95 nM. The addition of increasing concentrations of Enzalutamide effectively antagonised the DHT-mediated thermal stabilisation, with the highest Enzalutamide concentration, 100 µM, reducing the EC_50_ of DHT target engagement to 110 nM (Fig. 3a).

Our data demonstrates the action of Enzalutamide as a competitive antagonist and that the competition between agonist and antagonist observed should be amenable to the determination of an apparent Ki value. Hence, we derived the fold-shift in EC_50_ for agonist target engagement at each antagonist concentration relative to no added antagonist. The obtained EC_50_ fold-shift was then plotted against the antagonist concentration as a Schild plot analysis. A linear regression was fitted to enable calculation of pA_2_ and conversion to Ki. The determined Ki for the Enzalutamide-AR interaction was 309.7 nM (Fig. 3b). This value represents the intracellular equivalent of an apparent binding affinity as measured by inhibitory constant, Ki, and is determined using a cellular target engagement CETSA HT assay following compound incubation with prostate-cancer derived cells endogenously expressing AR. Both the receptor and test compounds in this system are entirely label-free, with the affinity information derived from these experiments solely a consequence of the intrinsic thermal stability of cellular AR and ligand-induced changes in thermal stability at 46 °C, as evidenced by an identical experiment performed in the absence of the 46 °C heat shock (Supplementary Fig. S3). We observed comparable competitive behaviour using an alternative AR-positive prostate cancer cell line, LNCaP, and determined a Ki value for the Enzalutamide-AR interaction within LNCaP cells in a similar range to the one measured in CWR22Pc-R1-AD1 cells (Supplementary Fig. S3).

To elucidate AR pharmacology, we applied the CETSA HT assay to determine apparent intracellular Ki for a panel of known AR antagonists. ARN-509, Hydroxyflutamide, Enzalutamide, Nilutamide and Bicalutamide returned reproducible values for intracellular binding affinity to AR spanning a range of potencies (Fig. 3c, Supplementary Fig. S8). The apparent affinities derived correlated well with historical observations for AR antagonists. For example, Enzalutamide has been reported to bind AR with 5–8 fold greater affinity than Bicalutamide using a radiolabelled DHT displacement assay^27^, with a similar assay suggesting ARN-509 bound with 7–10 fold greater affinity than Bicalutamide^45^. Our label-free approach confirmed an 8 fold shift in affinity between Enzalutamide and Bicalutamide, and a 12 fold difference between ARN-509 and Bicalutamide (Fig. 3c).

Finally, we explored whether the values derived are affected by the temperature of the applied heat shock (46 °C). Four temperatures were selected from the melt curve (Fig. 2a) where a suitable window between un-stabilised and agonist-stabilised AR allowed CETSA experiments. The selected temperatures; 44 °C, 46 °C, 48 °C and 50 °C, span a 6 °C range where a significant difference in determined Ki might be expected if it was influenced by the temperature of the heat shock. The competition experiment from Fig. 3a was repeated in parallel with heat shocks at these four temperatures, revealing comparable values for apparent Enzalutamide Ki ranging from 165.6 nM to 270.2 nM, with no clear correlation with temperature (Fig. 3d, Supplementary Fig. S9). As varying the temperature of the heat shock applied to the system had no effect on determined Ki, we do not believe the heat shock influences the determined intracellular affinity value. As an additional note, the DHT EC_50_ determined for ITDRF_CETSA_ experiments at these four temperatures did not dramatically differ (Supplementary Fig. S9). While other systems have shown varying ITDRF_CETSA_ EC_50_ at different temperatures, the use of a Schild plot, which uses the fold-change in EC_50_ relative to the control condition without antagonist added, should effectively normalise these differences and return the same apparent Ki value, thus reducing the temperature-subjectivity of CETSA experiments.

## Discussion

As a measure of cellular target engagement, CETSA offers unique benefits through application to physiologically relevant samples without the requirement for any modifications to either the target protein or the test compound. CETSA assesses engagement with the target in a setting where it is endogenously expressed and is not modified by any tags or reporter genes. The present comparative study demonstrates the advantages of CETSA HT assays for target-driven early drug discovery, where it provides a more precise measure of direct binding than functional cellular assays, in a more physiologically relevant manner than alternative cellular target engagement technologies^46,47^. Additionally, quantification of compound binding to AR has been limited by an inability to generate sufficient purified recombinant full-length protein^9,48^. CETSA therefore provides a feasable alternative to measure binding to a target where traditional biophysical methods are not possible, for example when recombinant protein cannot be produced.

Recent attempts to perform CETSA on AR did not demonstrate target engagement, although the tested compound was not a validated AR binder and was not expected to directly target AR^49^. Our application of CETSA Classics to AR revealed that target engagement of antagonist does not modulate AR thermal stability, while target engagement of agonist causes a thermal stabilisation. The thermal stability of AR is only altered when the ligand binding domain is occupied by an agonist. The reasons for this are not clear, although structural data suggests agonists and antagonists can occupy different regions of the ligand binding domain^32,50^. However, target engagement of antagonist was able to reverse agonist-mediated thermal stabilisation, presumably through direct competition of agonist from the ligand binding domain as competition experiments show responses typical of competitive binding (Fig. 3a). This demonstrates for the first time a system where CETSA can inform not only on target engagement, but on an element of binding mode, differentiating agonist and antagonist binders.

These observations were succesfully translated into a CETSA HT assay enabling screening for intracellular AR target engagement. This approach has only been reported for a few example proteins^26,29^, herein extended to an additional protein, AR, and a novel target class, nuclear hormone receptors. The differing profile of a range of inhibitors of AR co-regulators between functional assays measuring AR-driven transcription and CETSA HT emphasise the potential advantages of CETSA-based screening to report on direct binders of a target of interest. Measuring the transcriptional response of AR identifies inhibitors of AR co-regulators such as menin, TrkA, p23 and BRD4 as active compounds. Yet the CETSA HT technology provides evidence that these compounds do not bind to AR and for the purposes of direct AR antagonists can be considered “pathway” effects, detected as a consequence of measuring a downstream biological response. The use of CETSA HT rather than an alternative assay measuring a functional response can therefore increase the confidence that the test compound is directly binding to the target protein. Future application of CETSA HT for systems which show competitive destabilisation as with AR antagonists should control for changes in total protein, which can be achieved by parallel screening in the absence of a heat shock (Supplementary Fig. S6). CETSA HT technology may have immediate application for the identification of novel direct AR antagonists in a disease relevant context for the treatment of CRPC.

Previous studies have demonstrated the application of CETSA for ranking the potency of target engagement between different molecules^7,26,29^, but the measures returned have to date been considered relative potencies. Here we report, to our knowledge, the first application of CETSA to determine more quantitative apparent intracellular binding affinities. These experiments allow calculation of an apparent Ki value to quantify the intracellular ligand-receptor affinity as measured in live cells, whilst maintaining the aforementioned advantages of CETSA technology, namely a physiologically relevant, label-free system. While recent reports have demonstrated the ability to determine Ki using an alternative measure of target engagement in cellular systems^51^, this technology is limited by availability of a fluorescently-labelled probe compound and is yet to demonstrate comparable throughput.

The CETSA HT assay described herein should be highly informative for future studies into AR biology and pharmacology. Within the context of a CWR22Pc-R1-AD1 cell background, which exclusively expresses a structurally full-length AR containing a H874Y mutation^23^, the rank order of antagonist binding was determined as ARN-509 > Hydroxyflutamide > Enzalutamide > Nilutamide > Bicalutamide (Fig. 3c). Interestingly, the H874Y mutation has previously been reported to allow AR to utilise Hydroxyflutamide, but not Bicalutamide, as an agonist^12,52^. While we observed a more potent binding affinity for Hydroxyflutamide compared to Bicalutamide in the same mutational context, it did not yield an agonist-binding mode within our CETSA HT assays (i.e. Hydroxyflutamide alone did not thermally stabilise AR as observed with other AR agonists). Similarly, Hydroxyflutamide and other antagonists did not show any evidence of binding as an agonist in LNCaP cells harbouring the T877A mutation which has also been linked to paradoxical agonism^53,54^ (Supplementary Fig. S3b). It would be of interest to extend these binding studies to explore paradoxical AR activation using additional cell lines which harbour differing AR mutations. While the current system has been optimised and validated for antagonists of the Androgen Receptor, future work will look to extend this proof-of-principle study to explore its application to determine apparent intracellular binding affinity for additional intracellular protein targets.

## Methods

### Cell culture

CWR22Pc-R1-AD1 cells and LNCaP cells were cultured in RPMI1640 medium with L-glutamine (Sigma) supplemented with 10% foetal bovine serum (FBS) in a humidified incubator at 37 °C, 5% CO_2_. Cell banks were confirmed as mycoplasma free and passed STR fingerprint analysis.

### CETSA Classics

CETSA Classics experiments were performed according to the general CETSA protocol^7,26^ to establish melt curves and ligand induced shifts. Intact, viable cells (CWR22Pc-R1-AD1) were harvested and resuspended to a final concentration of 20 × 10^6^ cells/mL in Hank’s balanced salt solution (Thermo Scientific). Cells were divided into separate aliquots and treated with DMSO control, 100 nM DHT, 100 µM Enzalutamide or by a combination of Enzalutamide (30 or 100 µM) and 100 nM DHT. Following 1 hour incubation at 37 °C, the treated samples were aliquoted into a PCR plate (AB1400, Thermo Scientific) and heated at temperatures ranging from 37 to 63 °C for 3 minutes (Veriti thermal cycler, Thermo Scientific), before they were snap frozen in liquid nitrogen. All samples were supplemented with Complete protease inhibitor cocktail (Roche) before lysis by 3x freeze-thaw using liquid nitrogen and a water-bath. Samples were clarified by centrifugation at 20,000xg for 20 minutes, and thereafter 40 µl of each supernatant was collected and mixed with 20 μl gel loading buffer (NuPAGE LDS sample buffer, Thermo Scientific). Protein separation was done using NuPAGE 4–12% BisTris gels (Thermo Scientific), where 10 μl of each sample mixture was loaded per lane, and run in MOPS buffer (Thermo Scientific). Western blots were performed using an iBlot2 device (Thermo Scientific) on nitrocellulose membranes. Transfer was optimised at 25 V for 8 minutes. Blocking and dilution of antibodies were performed in 5% non-fat milk in Tris-Buffered Saline and TWEEN^TM^20. The mouse derived anti-AR antibody (AR441, Dako) was diluted to 1:1000 and incubated overnight at 4 °C. Specific protein bands of AR were detected using horseradish peroxidase (HRP) conjugated secondary antibody (sc-2055, Santa Cruz Biotechnology) together with Clarity Western ECL substrate (Bio-Rad Laboratories).

### Optimisation of AlphaScreen® antibody pair

The process for identification and optimisation of antibody pairs for quantification of AR by AlphaScreen^®^ is described in Supplementary Methods and Supplementary Fig. S2. Mouse derived anti-AR antibodies were from Dako (AR441, M356201-2) and BDBioscience (554225). Rabbit derived anti-AR antibodies were from Abcam (Ab200827), Santa Cruz (N-20, Sc-816) and MerckMillipore (PG-21, 06-680). Antibodies were diluted 1:1000 into 1x ImmunoAssay Buffer (PerkinElmer, AL000F) and 3 µL of combined antibody pairs added to 3 µL of CWR22Pc-R1-AD1 cell lysate prepared in 1x Alpha SureFire Ultra Lysis Buffer (PerkinElmer) in a ProxiPlate384 Plus plate (PerkinElmer, 6008280). Following 30 minute incubation, 3 µL of a combined solution of Anti-mouse IgG Alpha Donor beads (PerkinElmer, AS104D) at 120 µg/mL and Anti-rabbit IgG (Fc specific) AlphaLISA Acceptor beads (PerkinElmer, AL104C) at 30 µg/mL in 1x ImmunoAssay Buffer was added to yield a final donor concentration of 40 µg/mL and a final acceptor concentration of 10 µg/mL. Plates were incubated 16 h in the dark. Signal was analysed using an EnVision platereader (PerkinElmer). This process was repeated with variation of anti-AR antibody concentrations, cell density and AlphaScreen^®^ reagents to yield optimal signal, as described in Supplementary Fig. S2.

### CETSA HT melt curve

CWR22Pc-R1-AD1 cells were harvested and resuspended to 6.25 × 10^6^ cells/mL in complete media. Cells were split into separate pools and treated with DMSO control, 100 nM 5α-Dihydrotestosterone (Sigma, D-073-1ML) or 10 µM Enzalutamide before seeding 20 µL/tube into 24x MicroAmp^®^ 8-tube strips, 0.2 mL PCR tubes (ThermoFisher, N8010580). Samples were prepared such that each PCR strip contained 4x compound treated samples and 4x vector control treated samples, with each PCR strip subsequently being heat shocked at a single temperature. Following 2 h incubation under tissue culture conditions, heat shocks were performed at indicated temperatures for 3 minutes followed by a 3 minute cool at 25 °C using a Veriti SimpliAmp (ThermoFisher). Samples were stored at 4 °C until all heat shocks were complete before addition of 20 µL/tube 2x SureFire Lysis Buffer. Following 10 minute incubation, samples were briefly clarified. Samples were mixed by a 10 µL aspirate and dispense (note resuspension of any pellet is critical) and 3 µL of lysate was transferred to a 384w ProxiPlate. A solution of 1x ImmunoAssay Buffer was prepared containing 0.08 µg/mL Dako Mouse anti-AR antibody, 0.15 µg/mL MerckMillipore Rabbit anti-AR antibody, 120 µg/mL Anti-mouse IgG Alpha Donor beads and 30 µg/mL Anti-rabbit IgG AlphaLISA Acceptor beads. 6 µL of this solution was added under subdued light to the lysate in the ProxiPlate, sealed and incubated 16 h in the dark before analysis using an EnVision platereader.

### Isothermal dose response fingerprint experiments (ITDRF_CETSA_)

ITDRF_CETSA_ experiments were performed in a plate-based, automation-compatible assay setup, as described in Supplementary Fig. S2e. Compound dose responses were dispensed into a FrameStar 384w PCR plate (4titude, 4ti-0384/C) using either an Echo^®^ 555 acoustic dispensor or a hp Tecan D300e digital dispensor. Internal standard controls were included on each plate (Supplementary Fig. S10). CWR22Pc-R1-AD1 cells or LNCaP cells were harvested and resuspended to 1.75 × 10^7^ cells/mL. To measure AR antagonist binders, cells were supplemented with 1 nM DHT. 10 µL of cells were seeded per well of the PCR plate using a Multidrop Combi dispensor, covered with a plate lid, briefly centrifuged, and incubated under standard tissue culture conditions for 2 h or indicated timepoint. Heat shock was performed at 46 °C for 3 minutes, followed by a 1 minute cool at 20 °C, using a 384w LightCycler^®^480 II (Roche). Cells were lysed by addition of 10 µL/well 2x SureFire Lysis Buffer using a Multidrop Combi. Following 10 minute incubation at room temperature, the solution was mixed (10 × 7 µL aspirate and dispense) and 3 µL lysate was transferred to a 384w ProxiPlate using a Bravo liquid handling system (Agilent). Antibody and AlphaScreen reagent, prepared as above, were added by Multidrop Combi (6 µL/well). Plates were incubated in the dark 16 h and analysed using an EnVision platereader.

### AR-driven transcription ARE Luciferase reporter assay

CWR22Pc-R1-AD1 cells stably expressing ARE-firefly Luciferase vector pGL4.23 Luc2/miniP (vector sequences available on request) were maintained in T175 cell culture flasks. Cells were harvested and DHT added to 1 nM final concentration. 2 × 10^4^ cells/well were seeded in 15 µL into a 384w white luminescence plate pre-dosed with compound dose responses using an Echo^®^ 555 acoustic dispensor, and incubated 24 h. 15 µL 2x Steady-Glo^®^ reagent (Promega) was added per well and incubated 15 min in the dark before reading luminescence using an EnVision platereader. Experiments were performed in phenol red free RPMI media supplemented with 10% charcoal stripped FBS.

### Competition Ki experiments

Antagonist was dosed into a 384w PCR plate using a hp Tecan D300e digital dispensor. A 2X DHT dose response was prepared in media and 5 µL added per well on top of the antagonist in the 384w PCR plate. CWR22Pc-R1-AD1 cells or LNCaP cells were harvested to 3.5 × 10^7^ cells/mL and 5 µL/well seeded using a Multidrop Combi dispensor. Plates were incubated, heat shocked, and transferred to endpoint as described above. Data was plotted in GraphPad Prism 6 to fit a nonlinear regression and determine 50% effective concentration (EC_50_). The EC_50_ determined at each antagonist dose (A’) was normalised to the EC_50_ determined in the absence of antagonist (A) as [Log_10_ ((A′/A) − 1)] to derive the fold-shift in agonist EC_50_ at each antagonist dose. Fold shifts greater than 2-fold, yielding a positive value for [Log_10_ ((A′/A) − 1)], were plotted against the logarithm of the antagonist dose, and a linear trendline fitted using Prism 6. The X intercept when Y = 0 was determined as pA_2_, and anti-logged to derive Ki.

### Data availability

The authors declare that all data supporting the findings of this study are available within the article and Supplementary Information, or are available from corresponding authors upon request.

## Electronic supplementary material

Supplementary Information
