# Supplementary material for: Determining direct binders of the Androgen Receptor using a high-throughput Cellular Thermal Shift Assay
Source: Sci Rep. 2018 Jan 9;8:163. doi: 10.1038/s41598-017-18650-x (PMC5760633; doi:10.1038/s41598-017-18650-x)
Supplement: Supplementary file 1 — Supplementary Information [file 41598_2017_18650_MOESM1_ESM.pdf]

Determining direct binders of the Androgen Receptor using a high-throughput Cellular Thermal Shift Assay

Joseph Shaw[1]*, Mathew Leveridge[1], Charlotta Norling[4], Jakob Karén[4], Daniel Martinez Molina[4], Daniel O'Neill[1], James E. Dowling[2], Paul Davey[3], Suzanna Cowan[3], Michael Dabrowski[4], Martin Main[1] and Davide Gianni[1]*

[1]Discovery Sciences, Innovative Medicines and Early Development Biotech Unit, AstraZeneca, 310 Cambridge Science Park, Cambridge, UK.

[2]Oncology, Innovative Medicines and Early Development Biotech Unit, AstraZeneca, 35 Gatehouse Park, Waltham, MA, USA.

[3]Oncology, Innovative Medicines and Early Development Biotech Unit, AstraZeneca, 310 Cambridge Science Park, Cambridge, UK.

[4]Pelago Bioscience AB, 171 65 Solna, Sweden.

*Corresponding authors: Joseph Shaw (E: Joseph.shaw@astrazeneca.com; T: +441223393405) and Davide Gianni (E: Davide.Gianni@astrazeneca.com; T: +441223223350).

Supporting Information

Supplementary Methods

Western blot analysis of AR for cell line validation

CWR22Pc-R1-AD1 cells were lysed in 1x NuPAGE LDS sample buffer, (Thermo Scientific) and lysate equivalent to 6 x 10^4 cells (15 µL) was resolved by NuPAGE 3-8% Bis-Tris acetate gel (Novex, WG1603A) at 150V for 80 minutes in MES running buffer. Gels were transferred to nitrocellulose membranes using an iBlot apparatus (ThermoFisher) and incubated in TBS Odyssey Blocking Buffer (LI-COR) for 1h, RT. Primary antibodies were added in Blocking Buffer for 1h, RT, and washed with 0.05% TBST prior to addition of IRDye 800CW Donkey anti-Rabbit IgG (LI-COR, P/N 926-32213) at 1:10,000 for 1h, RT. Primary antibodies were from Dako (AR441, M356201-2, 1:75) and BD BioScience (554225, 1:500). Blots were imaged using an Odyssey LICOR scanner.

Mass spectrometry measurement of intracellular Enzalutamide

Intracellular Enzalutamide concentrations were determined by mass spectrometry[1-3]. CWR22Pc-R1-AD1 cells were treated with a range of Enzalutamide doses for 2 h. Cells were harvested and washed 3x in PBS to remove extracellular compound before resuspension in 300 µL PBS and lysis by 3 cycles of 20 second sonication followed by 20 second recovery on ice. Cell lysates were stored at -80˚C prior to analysis by LCMS utilising a Waters Xevo TSQ (WAA697) and an Acquity UPLC system from Waters consisting of sample manager (L12USM631G), Acquity PDA (K12UPD606A), Column Manager (K12CMP412G) and Binary Solvent Manager (L12BUR860M). The Waters Xevo was operated in positive ion Electrospray (ESI) mode to detect the parent ion with *m/z* 465.1. Alongside samples a calibration curve was analysed spiked with 10, 7.5, 5, 2.5, 1, 0.5, 0.1 µM Enzalutamide in PBS. Calibration curve chromatograms were extracted, smoothed, integrated and fitted with a linear regression to give a standard curve to enable calculation of Enzalutamide concentration in test samples. Across all experiments r2 was >0.990 and the mean error of the QC samples of 17%.

Generation of a CETSA HT assay by AlphaScreen®

To generate a high-throughput compatible CETSA assay, thermostable (soluble) AR was quantified using an AlphaScreen® endpoint, negating the need for physical seperation of the soluble thermostable and insoluble thermally aggregated AR populations, as previously described[4]. Thermostable AR was quantified using AlphaScreen® technology (PerkinElmer) whereby close proximity of a donor bead and an acceptor bead allow for transfer of a singlet

oxygen from the donor to the acceptor, exciting a fluorophore to emit light at 520-620 nm[5]. Two primary anti-AR antibodies derived from mouse and rabbit were applied to bind AR simultaneously at separate epitopes. Secondary AlphaScreen® antibodies were combined with the primary antibodies in the form of AlphaScreen® donor beads functionalised with antibodies recognising mouse antibody IgG (PerkinElmer AS104D), and AlphaScreen® acceptor beads functionalised with antibodies recognising rabbit antibody IgG (PerkinElmer AL104C). Productive AlphaScreen® signal will only be observed in the presence of all of the following: correctly folded, soluble AR; mouse-derived anti-AR antibody; rabbit-derived anti-AR antibody; anti-Mouse AlphaScreen® donor; anti-Rabbit AlphaScreen® acceptor.

AR functional RT-qPCR assay

Compound was dosed into a 384w plate (Corning) and CWR22Pc-R1-AD1 cells, supplemented with 1 nM DHT, were seeded at 1.5 x 10^4 cells/well in 25 µL volume. Phenol red free RPMI1640 media supplemented with 10% charcoal-stripped serum was used throughout. Following 48 h incubation, plates were imaged using a 10x optics with an IncuCyte ZOOM (Essen BioScience) to calculate cellular confluency. Media was removed using an ELX405 CW washer (BioTek) and 12 µL RealTime ready Cell Lysis Kit supplemented with RNAse inhibitor (Roche) was added per well. Cells were lysed by 8 minute incubation with repetitive 2 min shakes at 100 rpm followed by 2 min clarification at 300xg. RealTime ready RNA Virus Master Mix (Roche) was prepared following manufacturer's instructions, supplemented with primers and probes for FKBP5 (FAM, IDT Ref Seq # NM_001145776(3)) and β-Actin (VIC, IDT Ref Seq # NM_001101(1)). 4.5 µL/ well was added to a 384w PCR plate (4titude). Following mixing, 0.5 µL of lysate was transferred to the PCR plate using a Bravo liquid handling system (Agilent). PCR plates were sealed and analysed using a LightCycer®II 480 (Roche). Reverse transription was performed for 8 min at 50˚C before 30 sec denaturation at 95˚C. 40 PCR cycles were performed consisting of 30 sec at 95˚C, 20 sec at 60˚C, fluorescence acquisition, and 1 sec at 72˚C. Calculated Cq values were exported for analysis.

References

1 Gordon, L. J. *et al.* Direct Measurement of Intracellular Compound Concentration by RapidFire Mass Spectrometry Offers Insights into Cell Permeability. *J Biomol Screen* **21**, 156-164, doi:10.1177/1087057115604141 (2016).

2 Zhang, X., Wang, R., Piotrowski, M., Zhang, H. & Leach, K. L. Intracellular concentrations determine the cytotoxicity of adefovir, cidofovir and tenofovir. *Toxicol In Vitro* **29**, 251-258, doi:10.1016/j.tiv.2014.10.019 (2015).

3 Mateus, A., Matsson, P. & Artursson, P. Rapid measurement of intracellular unbound drug concentrations. *Mol Pharm* **10**, 2467-2478, doi:10.1021/mp4000822 (2013).

4 Almqvist, H. *et al.* CETSA screening identifies known and novel thymidylate synthase inhibitors and slow intracellular activation of 5-fluorouracil. *Nat Commun* **7**, 11040, doi:10.1038/ncomms11040 (2016).

5 Eglen, R. M. *et al.* The use of AlphaScreen technology in HTS: current status. *Curr Chem Genomics* **1**, 2-10, doi:10.2174/1875397300801010002 (2008).

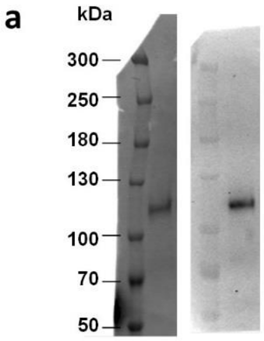

b

Incubated Conc (μM) 2 hr	Mean measured Conc n=3 (nM)
100	70
31.6	17
10	4
3.16	2 (n=1)

Supplementary Figure S1: Characterisation of CWR22Pc-R1-AD1 cells and intracellular measurement of Enzalutamide by mass spectrometry. **(a)** Western blot analysis of CWR22Pc-R1-AD1 cells for AR. Lysate from 6 x 10^4 cells was resolved by SDS-PAGE and western blot performed using Dako AR441 antibody (left) or BD BioScience 554225 antibody (right). Both identified a single AR band. **(b)** CWR22Pc-R1-AD1 cells were incubated 2 hours with indicated Enzalutamide doses, washed to remove extracellular compound and lysed by sonication. Cell lysates were analysed by LCMS alongside an Enzalutamide calibration curve to determine Enzalutamide concentration in each sample, confirming intracellular accumulation of Enzalutamide in a concentration dependant manner. Data is the mean of three biological replicates unless otherwise indicated.

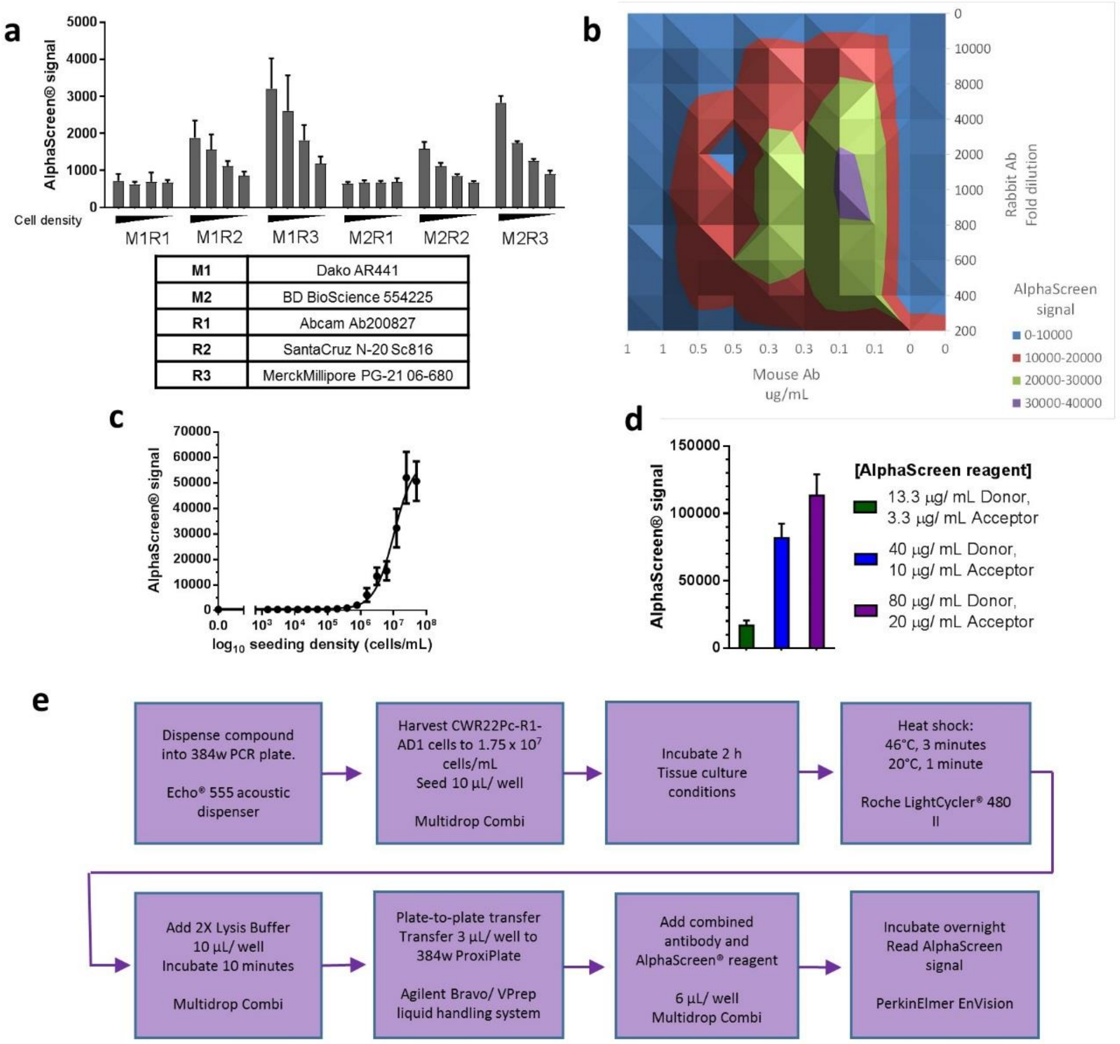

Supplementary Figure S2: Optimisation of an AlphaScreen® antibody pair for Androgen Receptor to develop a CETSA HT assay protocol. **(a)** Lysates from various cell densities of CWR22Pc-R1-AD1 probed with mouse- and rabbit-derived anti-AR antibody pairs in the presence of anti-mouse AlphaScreen® donor and anti-rabbit AlphaScreen® acceptor. The antibody pair M1R3 was selected. **(b)** Optimisation of AlphaScreen® signal by varying mouse anti-AR and rabbit anti-AR antibody concentrations at fixed cell density. Optimal primary mouse antibody was determined as < 0.1 µg/mL and subsequently further optimised to 0.08 µg/mL (data not shown). Optimal primary rabbit antibody was determined as 1:2000 (0.15 µg/mL). **(c)** Optimisation of AlphaScreen® signal by varying cell density. Cells were prepared to indicated density and 10 µL/well seeded prior to lysis, transfer and antibody addition. Optimal cell density was determined as 1.75 x 10^7 cells/mL (1.75 x 10^5 cells/well). **(d)** Optimisation of AlphaScreen® signal by varying donor and acceptor bead concentrations. Optimal donor was determined as a final concentration of 80 µg/mL and optimal acceptor was determined as a final concentration of 20 µg/mL. **(e)** Description of the 2-day, automation-compatible CETSA HT assay setup.

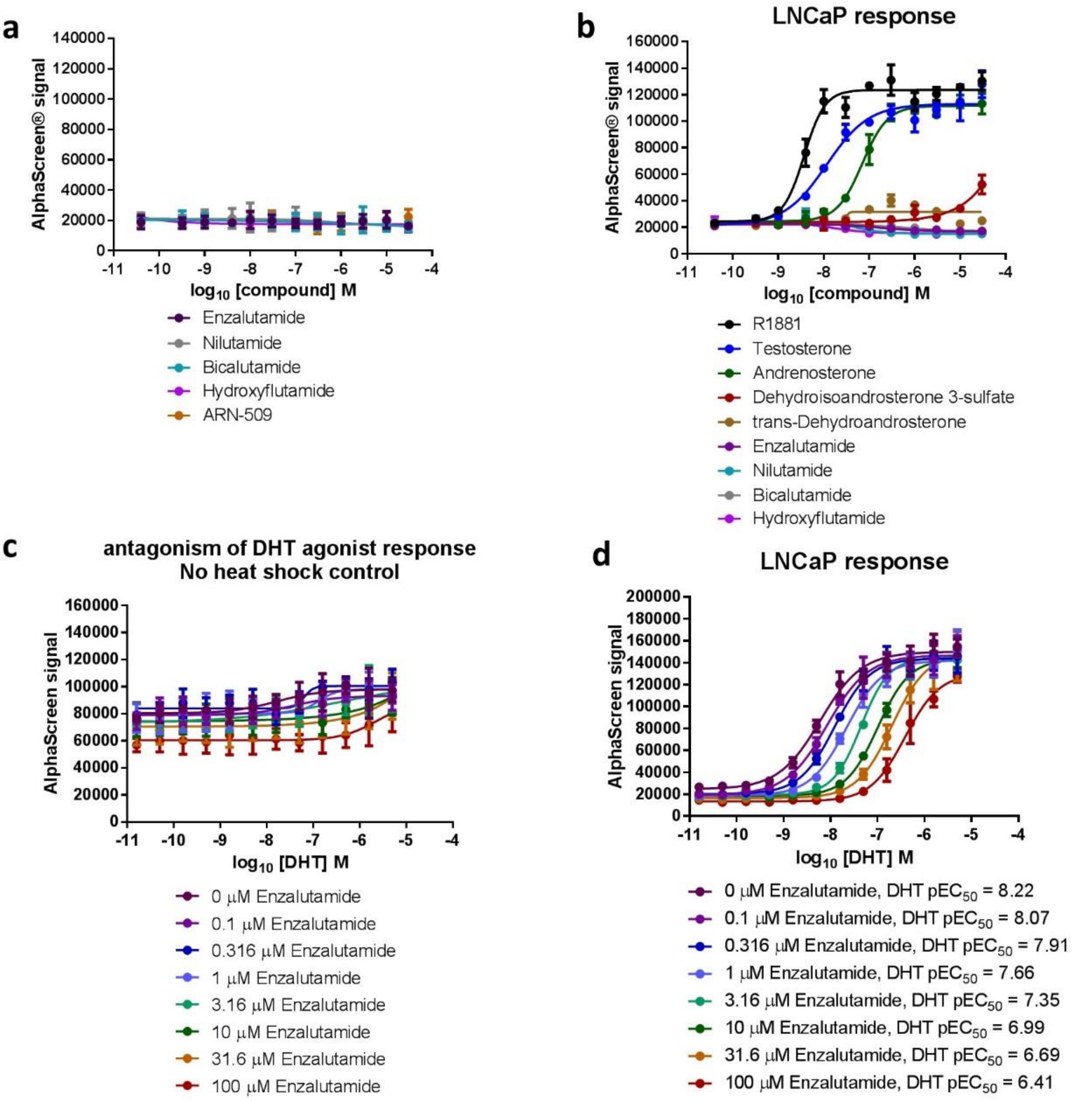

Supplementary Figure S3: Additional experiments for CETSA HT and Ki CETSA. **(a)** AR antagonists do not induce thermal stabilisation at 46˚C in CWR22Pc-R1-AD1 cells. Data the average of two technical replicates from the same experiments as data in Figure 2b. **(b)** Comparable observations were made for AR thermal stability using an alternative AR-positive cell line, LNCaP, where AR agonists induce thermal stabilisation upon target engagement, and AR antagonists do not. Testosterone EC_{50} = 11.6 nM, R1881 EC_{50} = 3.7 nM, Andrenosterone EC_{50} = 70.1 nM **(c)** An agonist : antagonist (DHT : Enzalutamide) competition experiment comparable to data in Figure 3a, performed in the absence of a heat shock, confirming that the AlphaScreen® CETSA HT quantification of AR response to agonism is mediated through the intrinsic thermal stability of cellular AR at 46°C. Data is the mean ± SD of n=4 and representative of two technical replicates. **(d)** An agonist : antagonist (DHT : Enzalutamide) competition experiment performed using an alternative AR-positive cell line, LNCaP, where Enzalutamide competitive antagonism of agonist target engagement (thermal stabilisation) is observed. Determined Enzalutamide Ki = 261.8 nM.

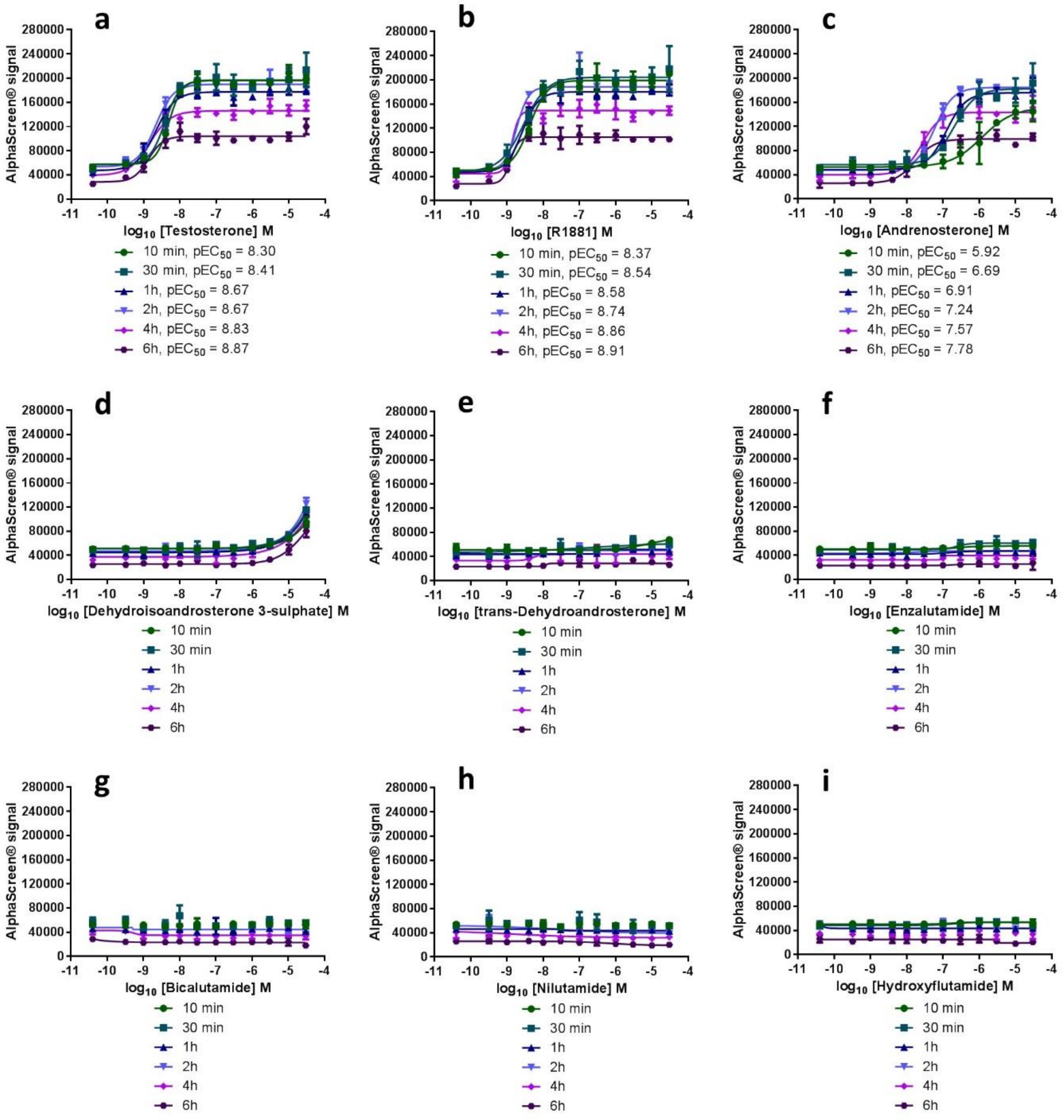

Supplementary Figure S4: Temporal analysis of target engagement by CETSA HT. $ITDRF_{CETSA}$ concentration response curves were performed following indicated length of compound incubation with CWR22Pc-R1-AD1 cells. Thermal stabilisation of AR was observed with **(a)** Testosterone and **(b)** R1881 within 10 minutes incubation, and was apparent up to 6 hours. AR thermal stabilisation by **(c)** Andrenosterone was incomplete at 10 minutes, requiring at least 30 minutes compound incubation, suggesting a requirement for intracellular conversion to the active AR binder. Partial stabilisation or no stabilisation were observed with **(d)** Dehydroisoandrosterone 3-sulphate and **(e)** trans-Dehydroandrosterone. No thermal stabilisation was observed at any point up to 6 hours with **(f)** Enzalutamide, **(g)** Bicalutamide, **(h)** Nilutamide or **(i)** Hydroxyflutamide, confirming lack of thermal stabilisation upon antagonist target engagement. Data the mean ± SD of n=3 from one technical replicate.

Supplementary Table S1: Raw data for comparative study for compound activity in an ARE-Luciferase reporter assay for AR-driven transcription and compound activity in CETSA HT. Compound effects on AR-driven transcription were measured as a reduction of luciferase under an Androgen Responsive Element (ARE) promoter within CWR22Pc-R1-AD1 cells stimulated with DHT (1 nM). Compound treatment was 24 h, data is the mean ± SD of two technical repeats. For selected compounds, effects on AR-driven transcription were also confirmed by RT-qPCR analysis (Supplementary Figure S5). Compound target engagement with AR was measured in CWR22Pc-R1-AD1 cells by CETSA HT as; i) the ability of a compound to compete out a fixed dose (1 nM) of DHT, and thus to induce thermal destabilisation at 46°C (black circles) as in Figure 2c; ii) The ability of a compound to influence thermal stability of AR at 46˚C in the absence of exogenous agonist (grey triangles) as in Figure 2b. Compound treatments were 2 h, data from one technical repeat. Selected compounds were also analysed in the absence of a heat shock to differentiate changes in AR thermal stability from changes in total cellular AR (Supplementary Figure S6).

Compound (Target)	CETSA HT response	ARE-Luciferase response	Conclusion
Enzalutamide (AR antagonist) 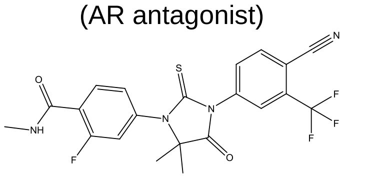	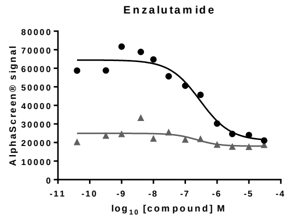 pIC50 = 6.54	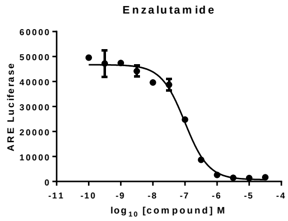 pIC_{50} = 7.00	Active in ARE-Luciferase assay<br><br>Active in CETSA HT
Hydroxyflutamide (AR antagonist) 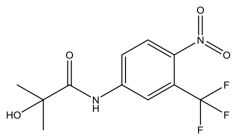	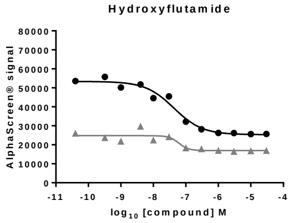 pIC50 = 7.34	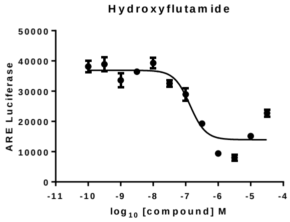 pIC_{50} = 6.87	Active in ARE-Luciferase assay<br><br>Active in CETSA HT
MK-2866 (AR antagonist) 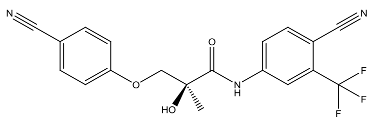	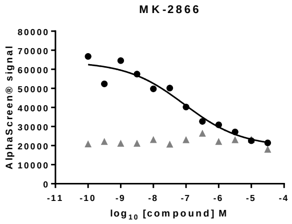 pIC_{50} = 7.05	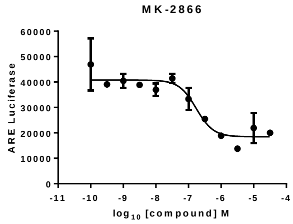 pIC_{50} = 6.77	Active in ARE-Luciferase assay<br><br>Active in CETSA HT
MI-136 (Menin) 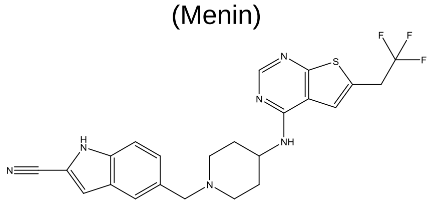	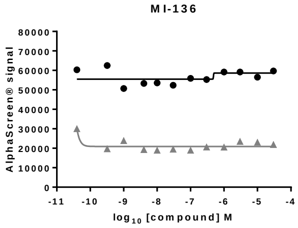 pIC_{50} = N.D.	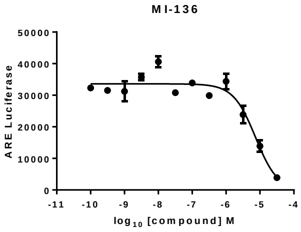 pIC_{50} = 5.13	Active in ARE-Luciferase assay<br><br>Inactive in CETSA HT

MI-503<br>(Menin)<br>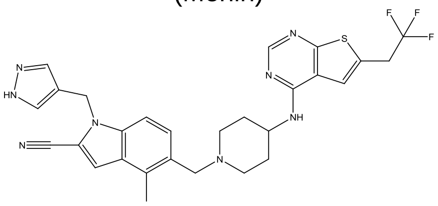	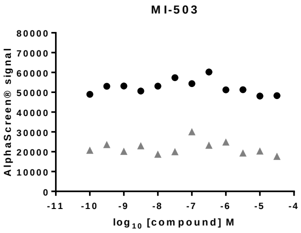<br>pIC$_{50}$ = N.D.	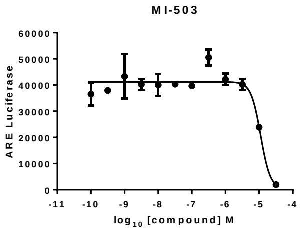<br>pIC$_{50}$ = 4.96	Active in ARE-Luciferase assay<br><br>Inactive in CETSA HT
Danusertib<br>(TrkA)<br>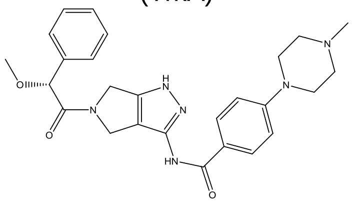	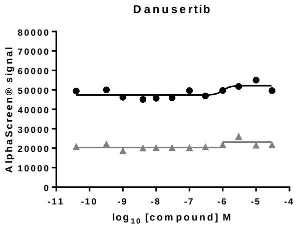<br>pIC$_{50}$ = N.D.	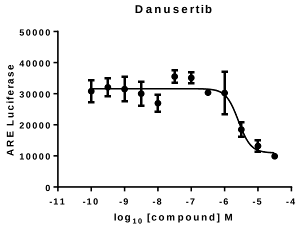<br>pIC$_{50}$ = 5.59	Active in ARE-Luciferase assay<br><br>Inactive in CETSA HT
Entrectinib<br>(TrkA)<br>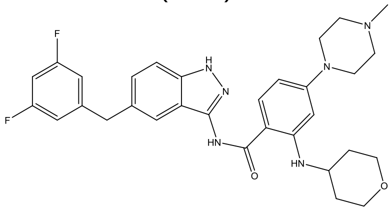	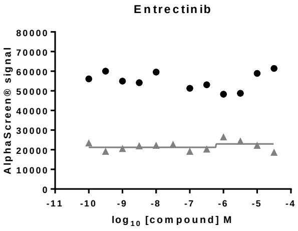<br>pIC$_{50}$ = N.D.	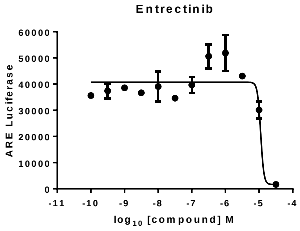<br>pIC$_{50}$ = 4.95	Active in ARE-Luciferase assay<br><br>Inactive in CETSA HT
Bayer pyrazolyl urea derivative inhibitor<br>(TrkA)<br>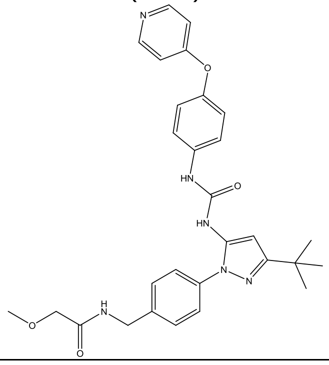	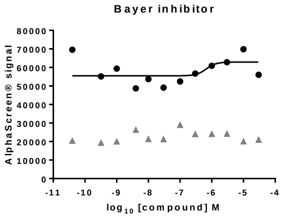<br>pIC$_{50}$ = N.D.	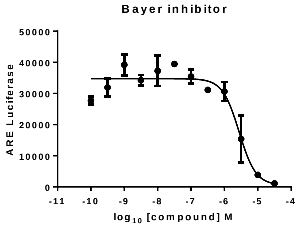<br>pIC$_{50}$ = 5.56	Active in ARE-Luciferase assay<br><br>Inactive in CETSA HT
JQ1<br>(BRD4)<br>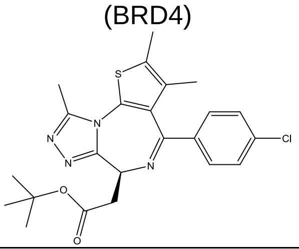	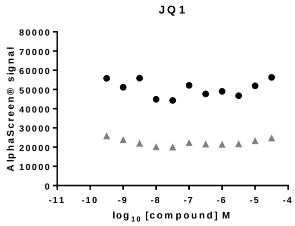<br>pIC$_{50}$ = N.D.	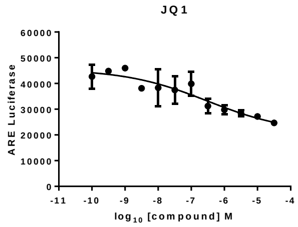<br>pIC$_{50}$ = 6.52	Partial active in ARE-Luciferase assay<br><br>Inactive in CETSA HT
OTX-015<br>(BRD4)<br>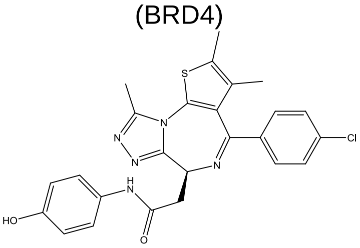	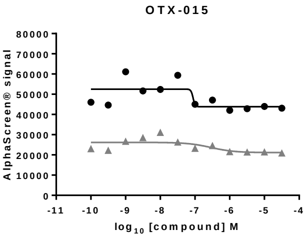<br>pIC$_{50}$ = N.D.	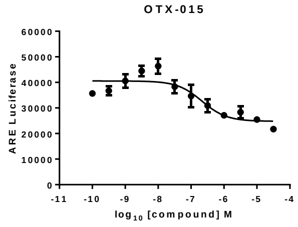<br>pIC$_{50}$ = 6.67	Partial active in ARE-Luciferase assay<br><br>Inactive in CETSA HT

Onalespib (Hsp90) 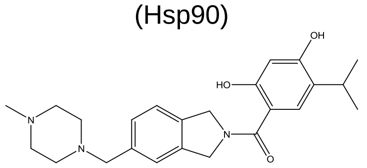	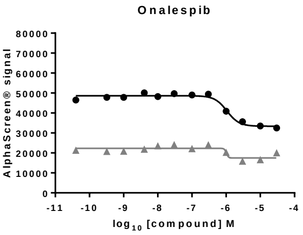 pIC50 = 5.98	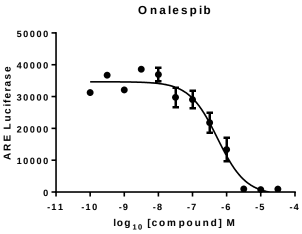 pIC_{50} = 6.27	Active in ARE-Luciferase assay<br><br>Active in CETSA HT
Tanespimycin (Hsp90) 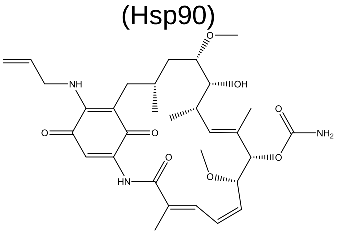	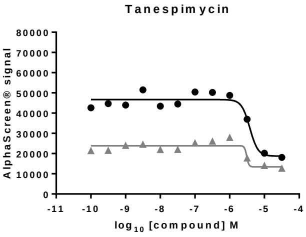 pIC_{50} = 5.42	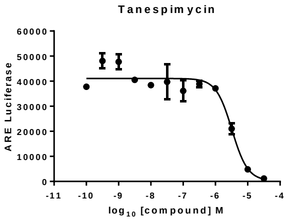 pIC_{50} = 5.49	Active in ARE-Luciferase assay<br><br>Active in CETSA HT
NVP-AUY-922 (Hsp90) 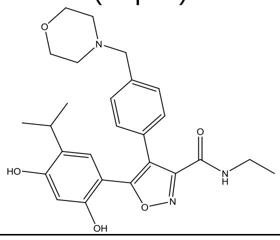	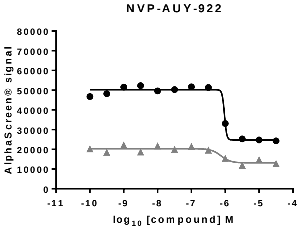 pIC_{50} = 6.02	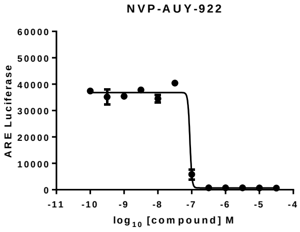 pIC_{50} = 7.05	Active in ARE-Luciferase assay<br><br>Active in CETSA HT
Niclosamide (AR downregulator) 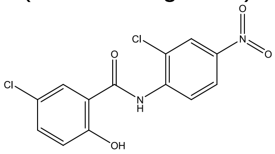	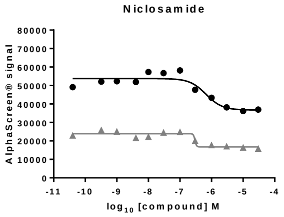 pIC50 = 6.19	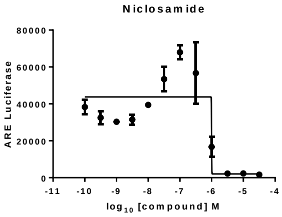 pIC_{50} = 6.02	Active in ARE-Luciferase assay<br><br>Active in CETSA HT
ACS-J9 (AR downregulator) 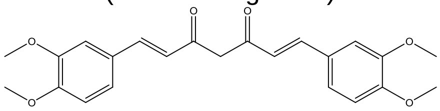	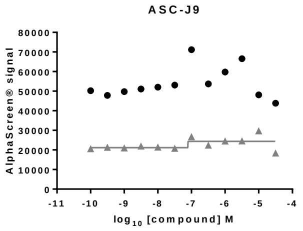 pIC_{50} = N.D.	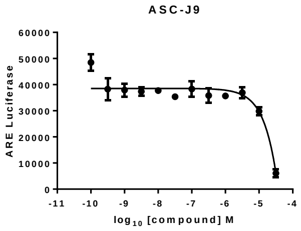 pIC_{50} = > 5.0	Active in ARE-Luciferase assay<br><br>Inactive in CETSA HT
Ailanthone (p23) 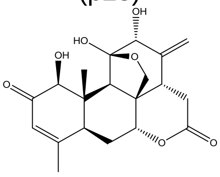	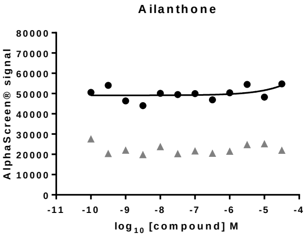 pIC_{50} = N.D.	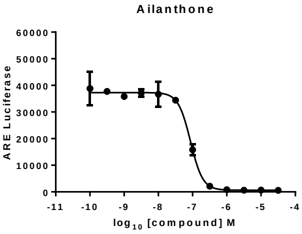 pIC_{50} = 7.06	Active in ARE-Luciferase assay<br><br>Inactive in CETSA HT

WNT-974<br>(WNT pathway, Porcupine)<br>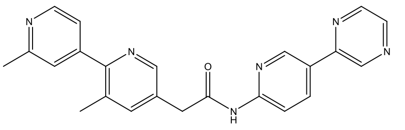	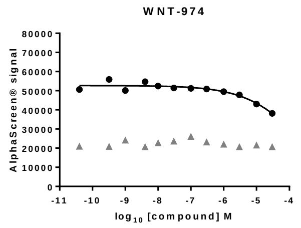<br>partial pIC50 = 3.62	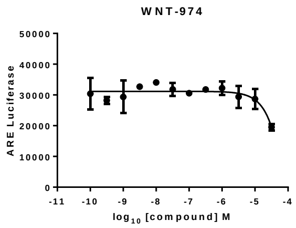<br>partial pIC_{50} = 3.19	Inactive in ARE-Luciferase assay<br><br>Inactive in CETSA HT
XAV-939<br>(WNT pathway, Tankyrase)<br>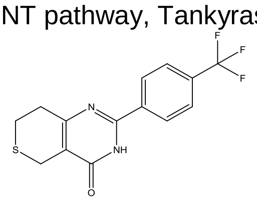	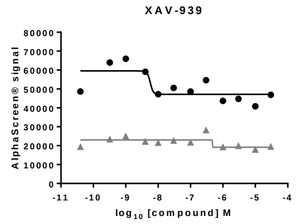<br>pIC_{50} = N.D.	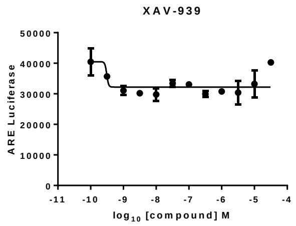<br>pIC_{50} = N.D.	Inactive in ARE-Luciferase assay<br><br>Inactive in CETSA HT
GSK-126<br>(EZH2)<br>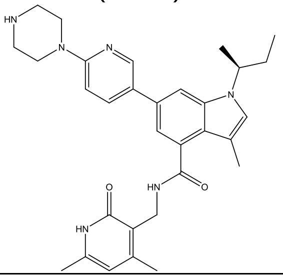	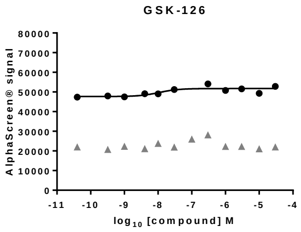<br>pIC_{50} = N.D.	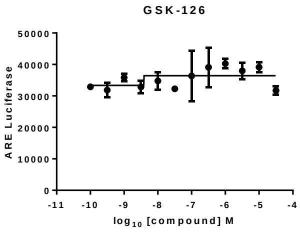<br>pIC_{50} = N.D.	Inactive in ARE-Luciferase assay<br><br>Inactive in CETSA HT
EPZ-6438<br>(EZH2)<br>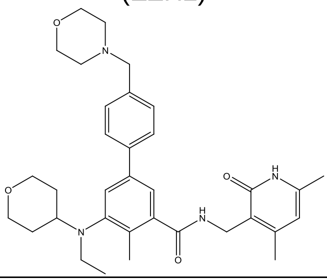	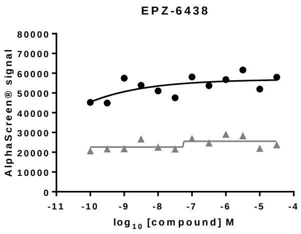<br>pIC_{50} = N.D.	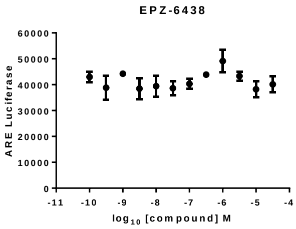<br>pIC_{50} = N.D.	Inactive in ARE-Luciferase assay<br><br>Inactive in CETSA HT
EED-226<br>(EZH2-EED)<br>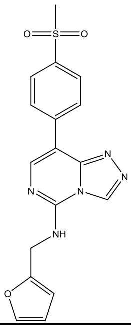	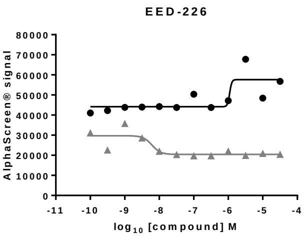<br>pIC_{50} = N.D.	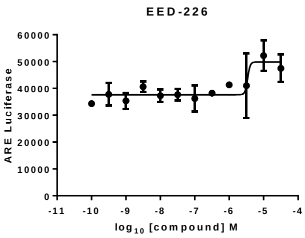<br>pIC_{50} = N.D.	Inactive in ARE-Luciferase assay<br><br>Inactive in CETSA HT

A-395<br>(EZH2-EED)<br>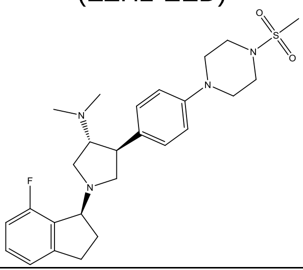	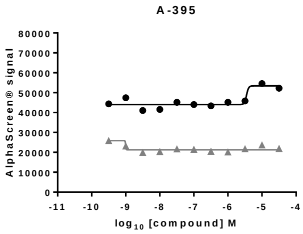 pIC$_{50}$ = N.D.	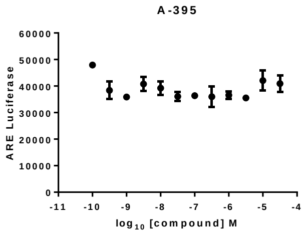 pIC$_{50}$ = N.D.	Inactive in ARE-Luciferase assay<br><br>Inactive in CETSA HT
GSK8814<br>(ATAD2)<br>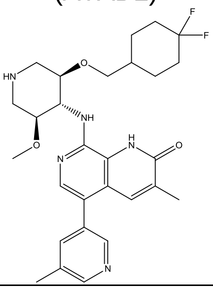	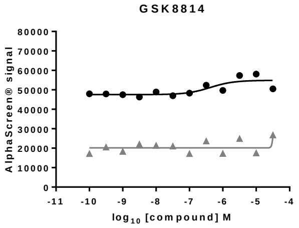 pIC$_{50}$ = N.D.	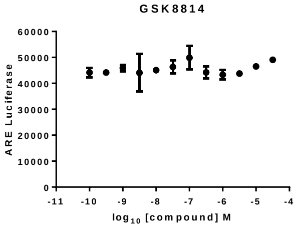 pIC$_{50}$ = N.D.	Inactive in ARE-Luciferase assay<br><br>Inactive in CETSA HT
GSK2879522<br>(LSD1)<br>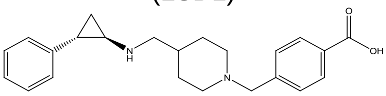	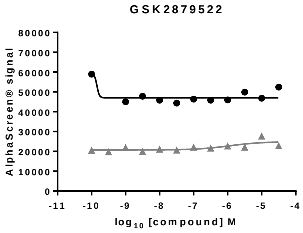 pIC$_{50}$ = N.D.	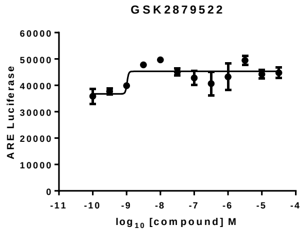 pIC$_{50}$ = N.D.	Inactive in ARE-Luciferase assay<br><br>Inactive in CETSA HT
Abiraterone<br>(CYP17A)<br>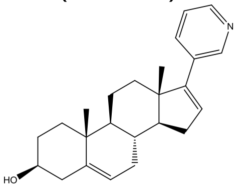	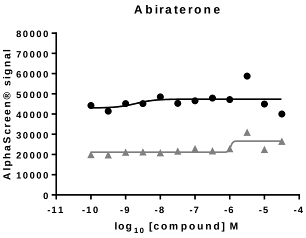 pIC$_{50}$ = N.D.	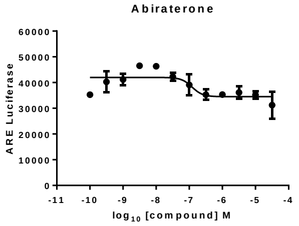 pIC$_{50}$ = N.D.	Inactive in ARE-Luciferase assay<br><br>Inactive in CETSA HT

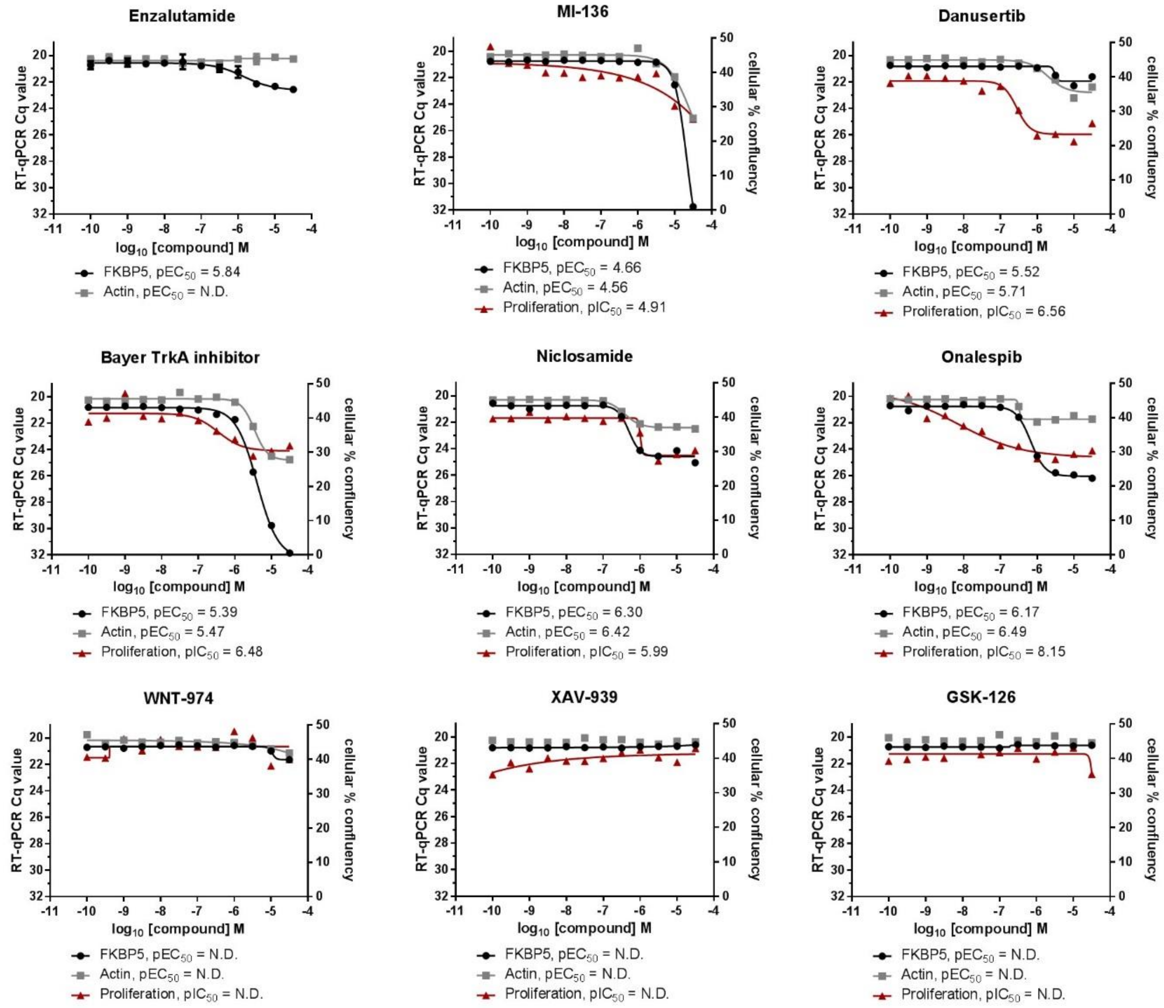

Supplementary Figure S5: Additional data for AR-driven transcriptional responses. Selected compounds from the pharmacology training set were analysed for reduction in mRNA levels of an AR-responsive transcript FKBP5 within CWR22-Pc-R1-AD1 cells, measured as an increase in Cq value by RT-qPCR and multiplexed with a β-Actin housekeeping gene. A measure of cell viability and proliferation of the same cells was performed by quantifying confluency using an IncuCyte ZOOM (plates imaged immediately before RT-qPCR analysis). Compound treatment was 48 h, data from one technical repeat.

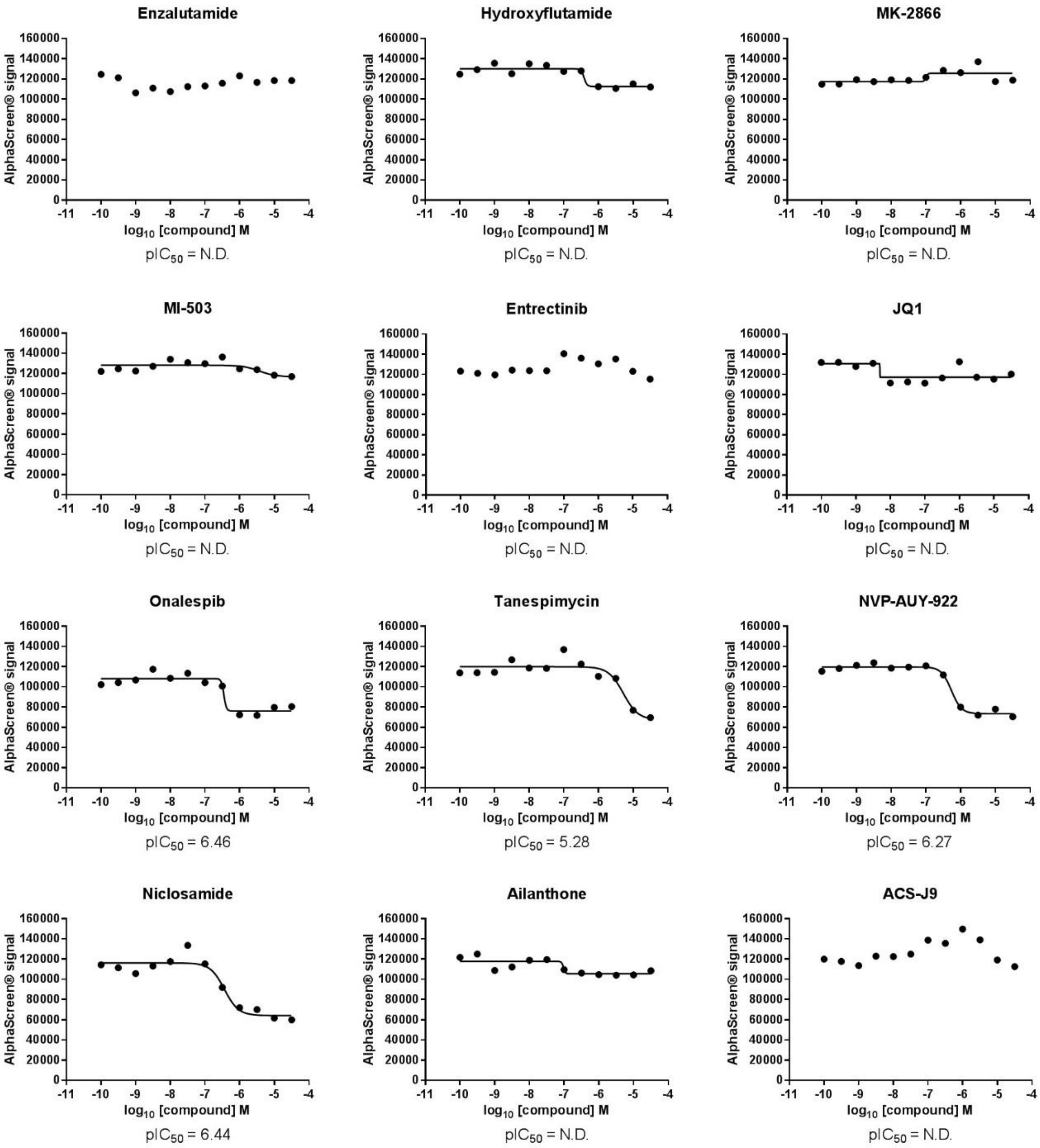

Supplementary Figure S6: CETSA HT controls in the absence of heat-shock to differentiate AR target engagement from AR degradation. Selected compounds were tested in the CETSA HT assay in the absence of exogenous DHT and without the 46˚C heat shock. In this format the assay essentially measures total AR following 2 h compound incubation. Data from one technical repeat, N.D. = not determined. AR antagonists Enzalutamide, Hydroxyflutamide and MK-2866 thermally destabilise AR as a consequence of target engagement (Supplementary Table S1), but do not reduce total AR. Niclosamide reduces total AR, as do the Hsp90 inhibitors Onalespib, Tanespimycin and NVP-AUY-922. The Menin inhibitor MI-503, the TrkA kinase inhibitor Entrectinib, the BRD4 inhibitor JQ1, the p23 inhibitor Ailanthone and the AR degrader ACS-J9 do not reduce total AR within 2 hours.

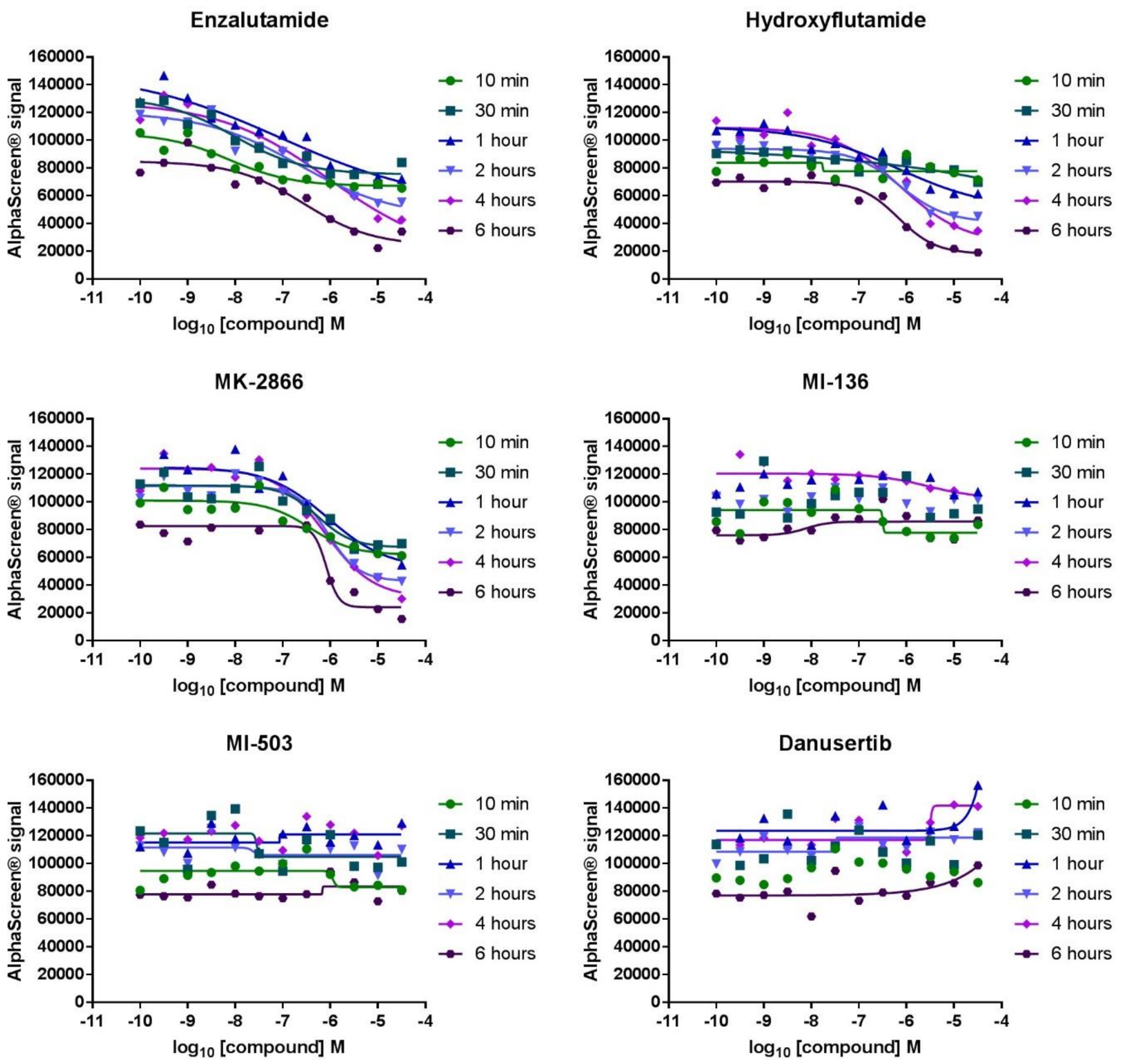
Enzalutamide
Hydroxyflutamide
MK-2866
MI-136
MI-503
Danusertib
AlphaScreen® signal
log10 [compound] M
10 min
30 min
1 hour
2 hours
4 hours
6 hours

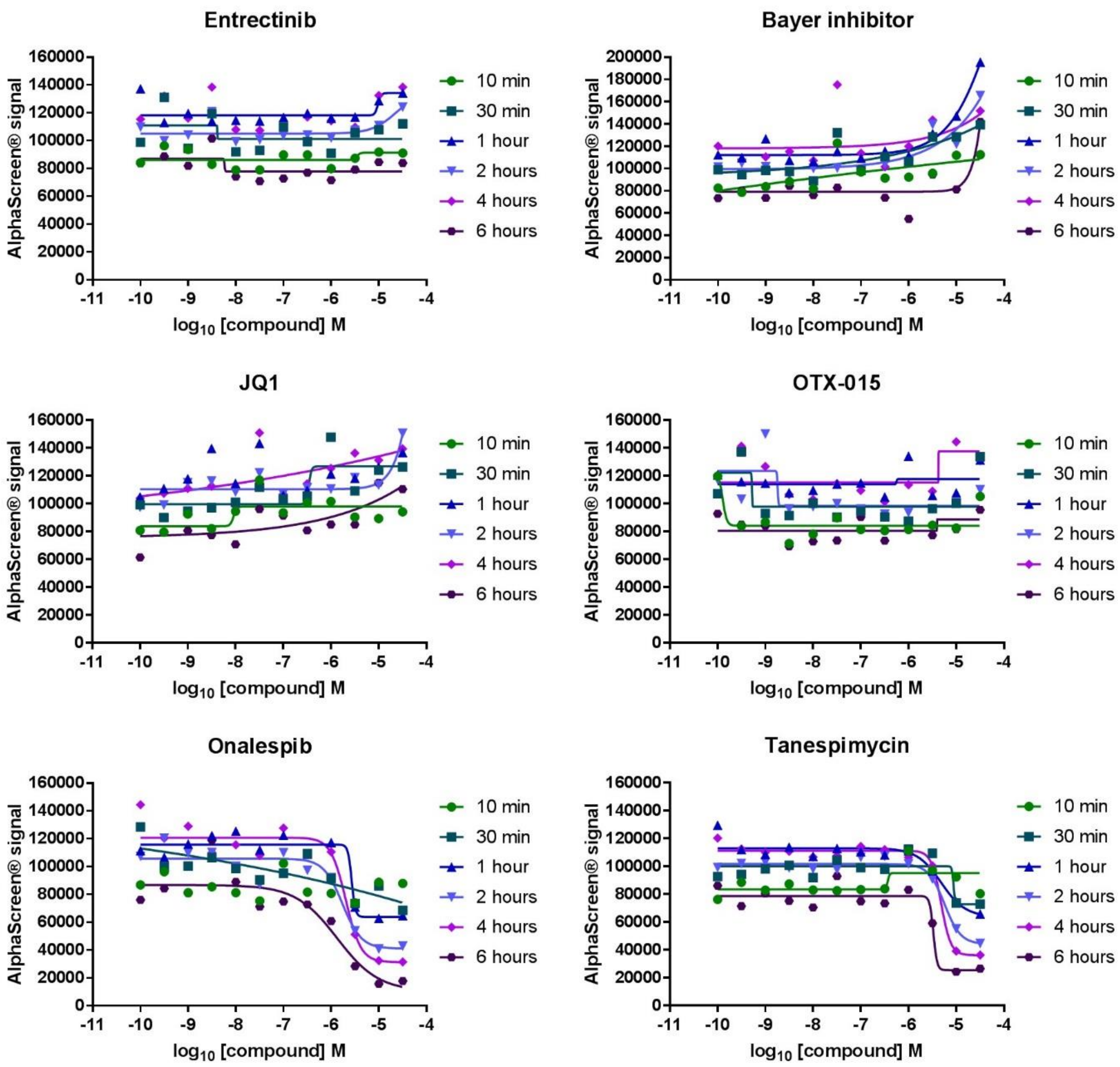
Entrectinib
Bayer inhibitor
JQ1
OTX-015
Onalespib
Tanespimycin
AlphaScreen® signal
log10 [compound] M
10 min
30 min
1 hour
2 hours
4 hours
6 hours

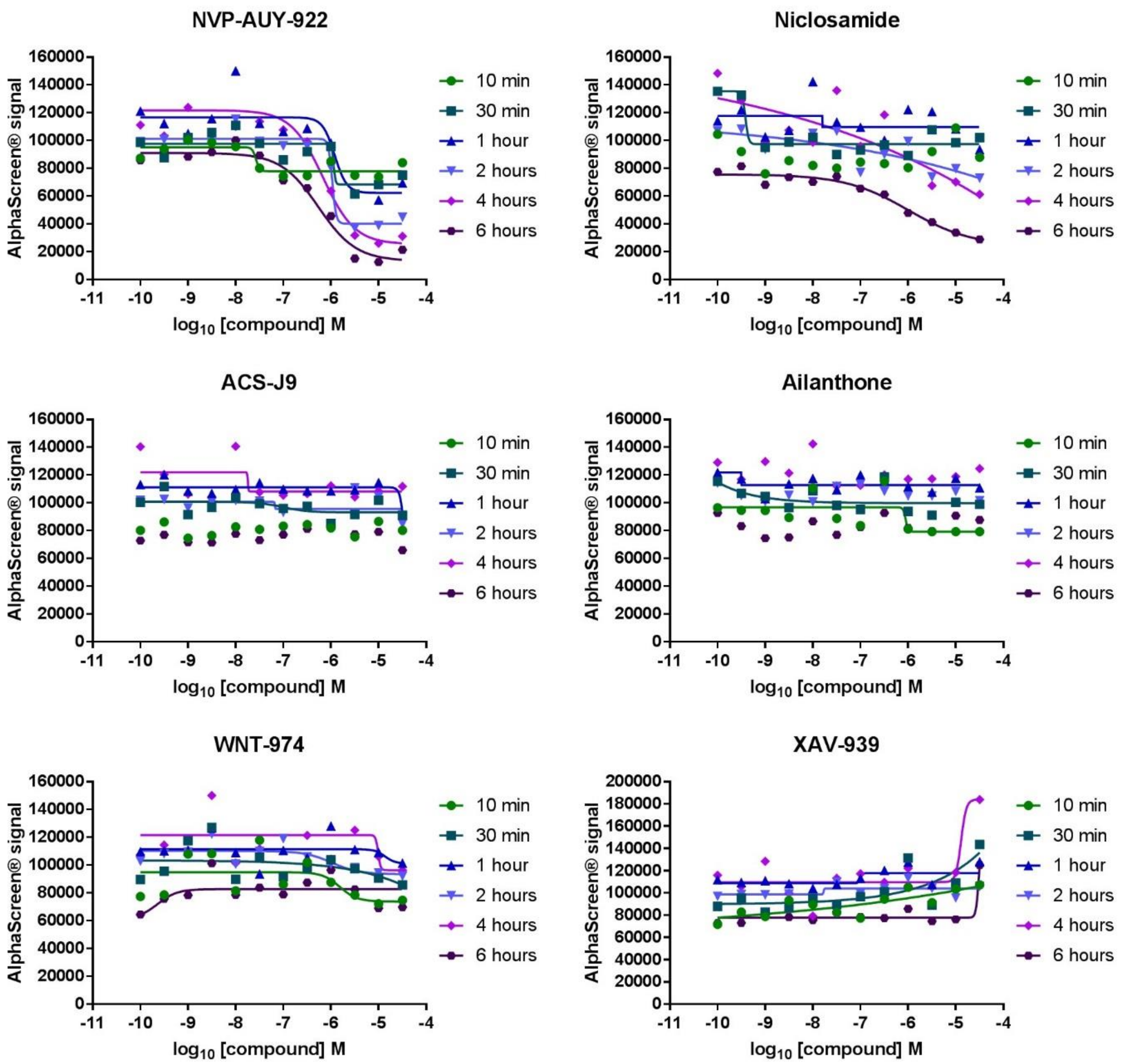
NVP-AUY-922
Niclosamide
ACS-J9
Ailanthone
WNT-974
XAV-939
AlphaScreen® signal
log10 [compound] M
10 min
30 min
1 hour
2 hours
4 hours
6 hours

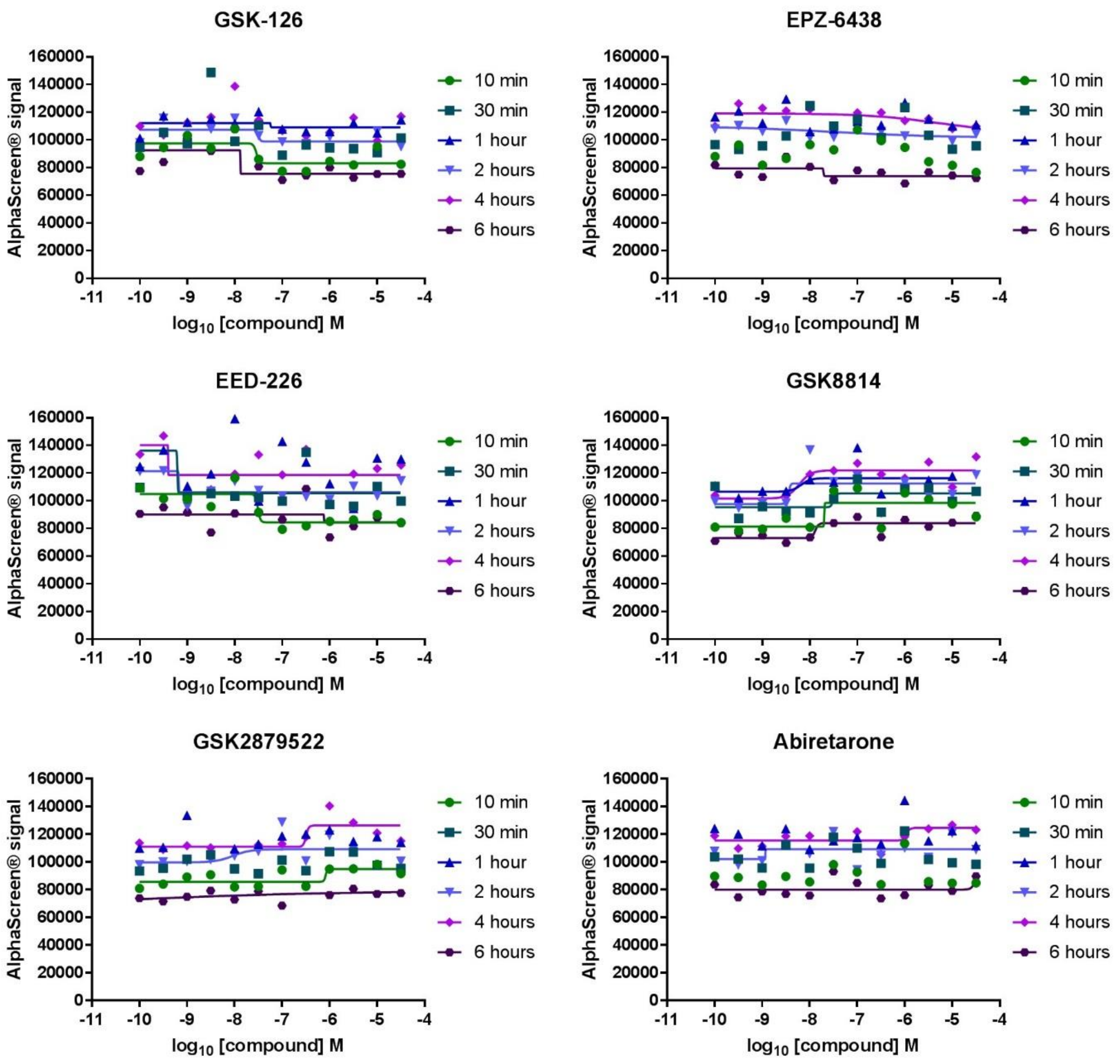

Supplementary Figure S7: Temporal analysis of target engagement by CETSA HT. Compounds from the pharmacology training set were screened by CETSA HT in the presence of 1 nM DHT with compound incubation for indicated timepoints between 10 minutes – 6 hours. Compounds which were inactive for CETSA HT target engagement following 2 hour incubation (Supplementary Table S1) were also inactive following 10 minutes to 6 hours incubation, confirming lack of target engagement. Known AR antagonists Enzalutamide, Hydroxyflutamide and MK-2866 showed increasing competition of DHT from AR over time as evidence of target engagement. Hsp90 inhibitors and Niclosamide showed varying temporal effects on AR AlphaScreen® signal which reflect the kinetics of AR degradation rather than target engagement (Supplementary Figure S6).

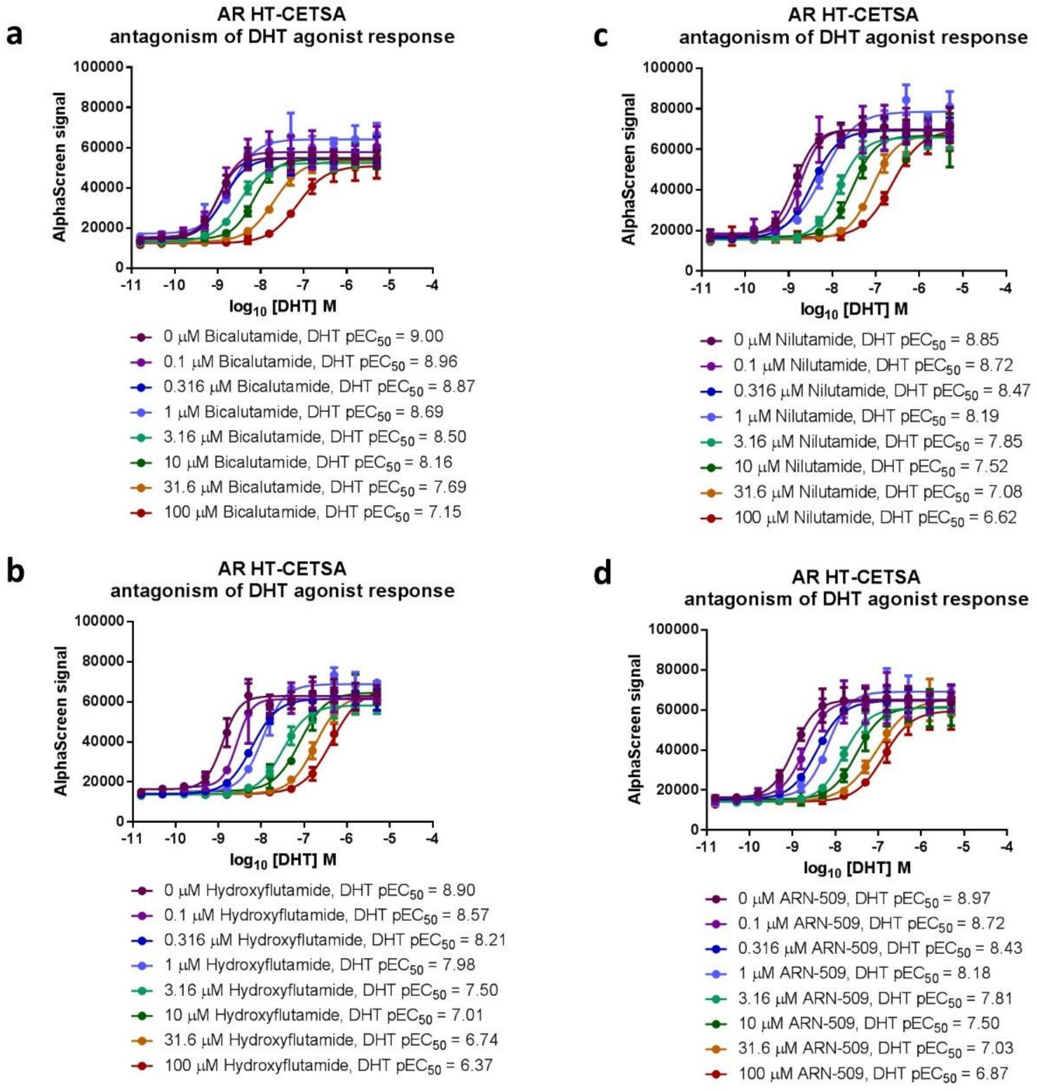

Supplementray Figure S8: Raw data for AR antagonist pharmacology intracellular Ki. CETSA HT was used to derive apparent intracellular Ki for the AR antagonists **(a)** Bicalutamide, **(b)** Hydroxyflutamide, **(c)** Nilutamide, **(d)** ARN-509. Data is the mean ± SD of n=4 and representative of three technical repeats, with determined Ki values reported in Figure 3c.

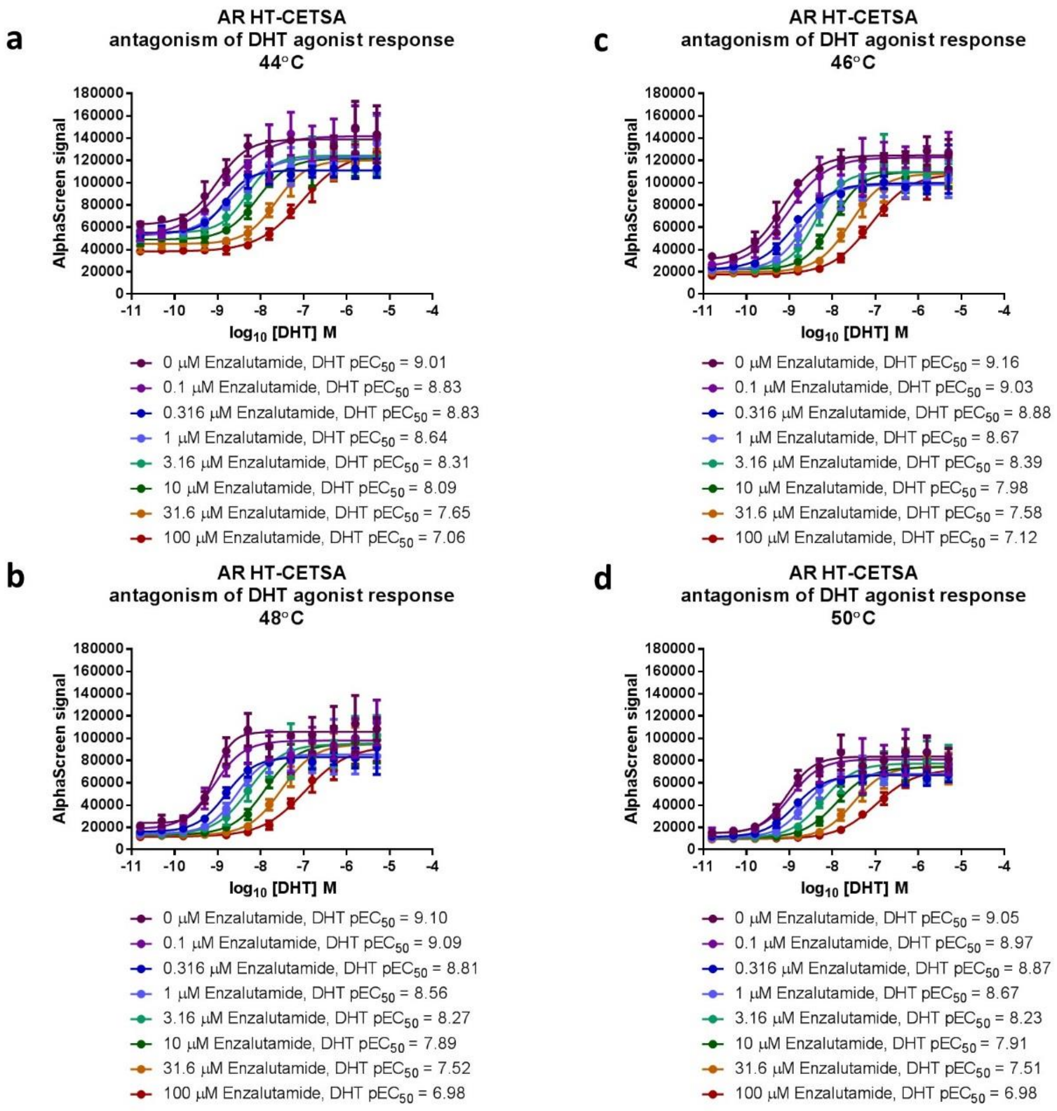

Supplementary Figure S9: ITDRF$_{CETSA}$ competition experiments across various heat shock temperatures. **(a)** Heat shock at 44˚C. **(b)** Heat shock at 46˚C. **(c)** Heat shock at 48˚C. **(d)** Heat shock at 50˚C. Data is the mean ± SD of n=4 samples and representative of three technical repeats.

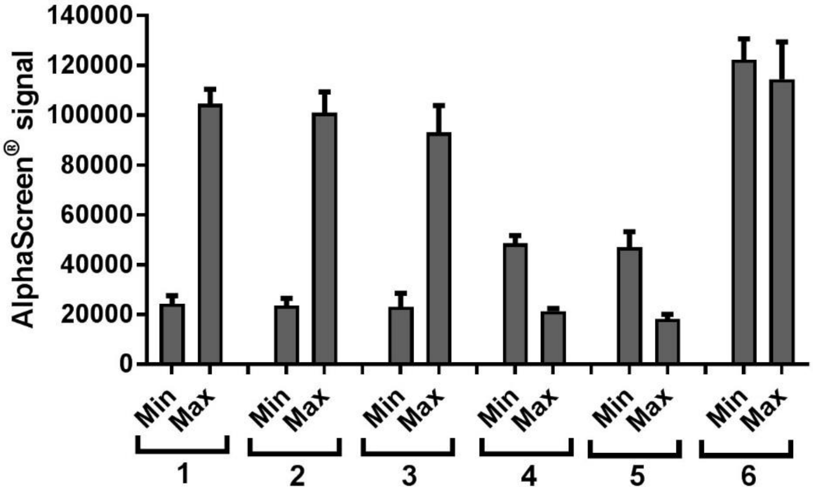

Plate	RZ′
Plate 1<br>AR agonist CETSA HT	RZ' = 0.74
Plate 2<br>AR agonist CETSA HT	RZ' = 0.69
Plate 3<br>AR agonist CETSA HT	RZ' = 0.71
Plate 4<br>AR antagonist CETSA HT	RZ' = 0.62
Plate 5<br>AR antagonist CETSA HT	RZ' = 0.54

Supplementary Figure S10: Internal standard controls and assay performance for CETSA HT screening plates. Plates 1 and 2; CETSA HT assays run in agonist mode (Figure 2a), internal standards were 30 µM R1881 (Max) and 0.3% DMSO (Min), n=12. Plate 3; CETSA HT screening of the AR pharmacology training set in agonist mode (Supplementary Table S1), internal standards were 30 µM R1881 (Max) and 0.3% DMSO (Min), n=12. Plate 4; CETSA HT assay run in antagonist mode in the presence of 1 nM DHT (Figure 2c), internal standards were 30 µM Enzalutamide (Max) and 0.3% DMSO (Min), n=16. Plate 5; CETSA HT screening of the AR pharmacology training set in antagonist mode (Table 1, Supplementary Table S1), internal standards were 30 µM Enzalutamide (Max) and 0.3% DMSO (Min), n=12. Plate 6; CETSA HT agonist mode internal standards 30 µM R1881 (Max) and 0.3% DMSO (Min) from the no heat-shock control plate for pharmacology training set screening, demonstrating controls do not affect AR signal in the absence of a 46°C heat-shock.

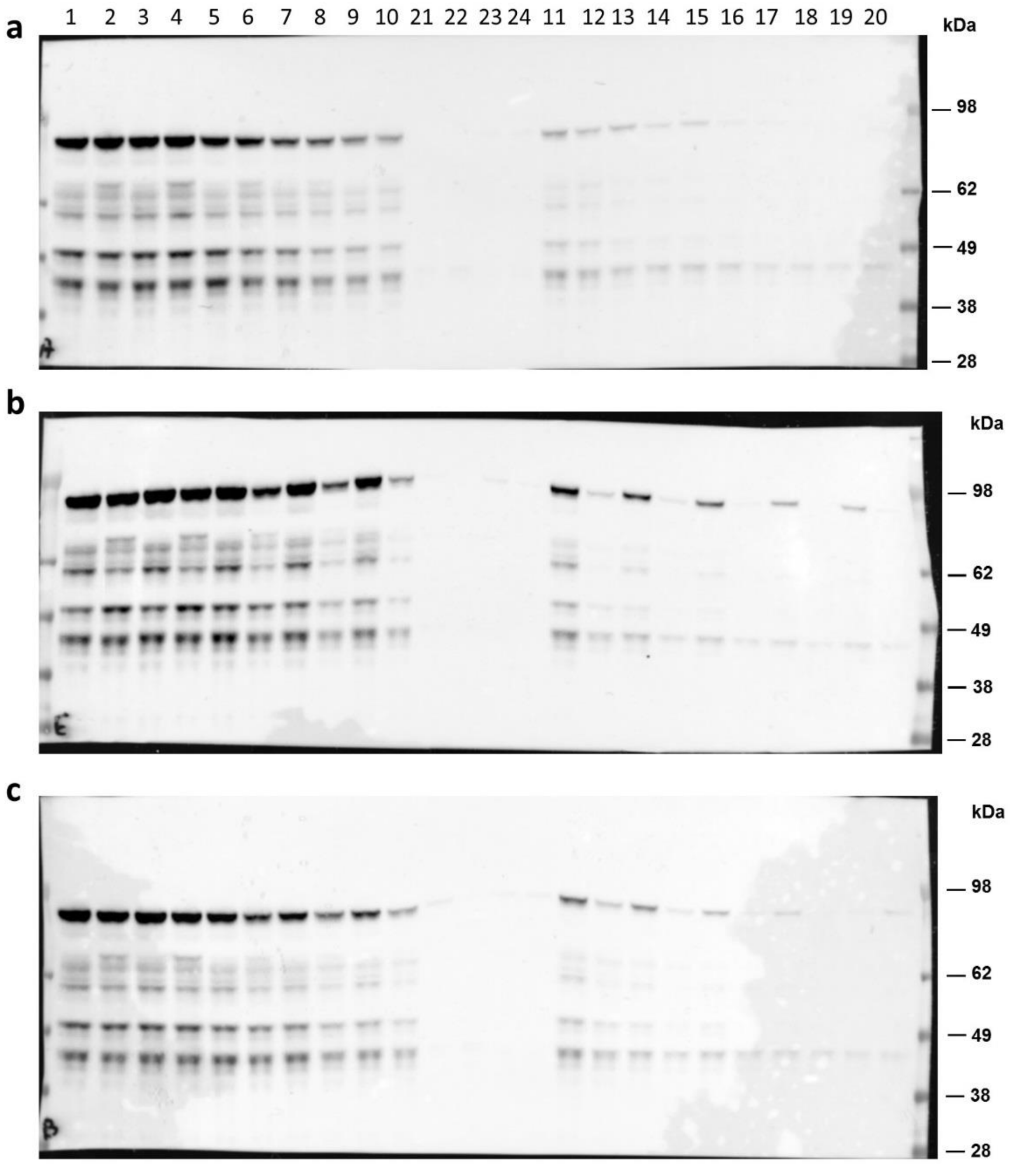

Supplementary Figure S11: Raw western blot images from Figure 1. **(a)** treatment +/- 100 µM Enzalutamide, **(b)** treatment +/- 0.1 µM DHT, **(c)** treatment +/- 30 µM Enzalutamide and 0.1 µM DHT. Molecular weight maker was See Blue Plus2 (ThermoFisher). The band corresponding to full length AR was quantified. Lanes contained the following samples; 1) vehicle, 37°C; 2) compound, 37°C; 3) vehicle, 40°C; 4) compound, 40°C; 5) vehicle, 43°C; 6) compound, 43°C; 7) vehicle, 45°C; 8) compound, 45°C; 9) vehicle, 47°C; 10) compound, 47°C; 11) vehicle, 49°C; 12) compound, 49°C; 13) vehicle, 51°C; 14) compound, 51°C; 15) vehicle, 53°C; 16) compound, 53°C; 17) vehicle, 55°C; 18) compound, 55°C; 19) vehicle, 57°C; 20) compound, 57°C; 21) vehicle, 60°C; 22) compound, 60°C; 23) vehicle, 63°C; 24) compound, 63°C.
